# An efficient mRNA delivery system for genome editing in plants

**DOI:** 10.1111/pbi.14591

**Published:** 2025-02-10

**Authors:** Fengti Qiu, Chenxiao Xue, Jinxing Liu, Boshu Li, Qiang Gao, Ronghong Liang, Kunling Chen, Caixia Gao

**Affiliations:** ^1^ Center for Genome Editing, Institute of Genetics and Developmental Biology Chinese Academy of Sciences Beijing China; ^2^ College of Advanced Agricultural Sciences University of Chinese Academy of Sciences Beijing China; ^3^ Qi Biodesign Life Science Park Beijing China; ^4^ New Cornerstone Science Laboratory Beijing China

**Keywords:** genome editing, DNA‐free, mRNA delivery, particle bombardment

## Abstract

Transgene‐free genome editing is important for crop improvement as it reduces unanticipated genomic changes. While mRNA delivery systems offer a powerful method for achieving transgene‐free genome editing, they remain inefficient and challenging in plants. Here we describe an efficient mRNA delivery system for plants with substantially improved editing efficiency. By optimizing the 5′ untranslated regions (5′UTRs) and poly(A) tails of *in vitro*‐transcribed (IVT) mRNAs and coating the mRNA with protamine during particle bombardment, we have developed an optimized mRNA delivery system termed v2_TMV/DEN2. This system enhanced the efficiencies of knock‐out, A‐to‐G and C‐to‐T base editing by an average 4.7‐, 3.4‐ and 2.5‐fold at various endogenous sites compared with plasmid‐based transient delivery system via particle bombardment in rice suspension cells and wheat immature embryos 48 h post‐transformation. Furthermore, we obtained edited plants with efficiencies of 5.0–180.8% and 26.1–26.2% using v2_TMV/DEN2 in rice and wheat, respectively, compared with 0.0–43.2% and 4.7–10.4% using plasmids. Our study provides a convenient and efficient mRNA delivery system for transgene‐free genome editing in plants.

## Introduction

CRISPR‐Cas genome editing technology has revolutionized agriculture over the past decade (Chen *et al*., 2019). Conventional plant transformation technologies deliver gene editing systems in the form of DNA, which causes random integration of pieces of DNA, and sustained intracellular expression of CRISPR/Cas systems can result in off‐target editing (Gao, 2021; Li *et al*., 2024). Owing to regulatory concerns and consumer resistance to transgenic crops, it is necessary to remove the transgene from transformed plants, which is a labour‐intensive and time‐consuming process (Gao, 2021). Transgene‐free gene editing systems avoid these problems. Such systems can involve transient expression of genome editing components based on DNA vectors using *Agrobacterium* or particle bombardment‐mediated transformation (Huang *et al*., 2023; Zhang *et al*., 2016). A more direct approach to producing transgene‐free genome‐edited plants is to deliver CRISPR components as mRNA or ribonucleoprotein (RNP). CRISPR‐Cas9 RNPs have been delivered into lettuce protoplasts (Woo *et al*., 2015), rice callus (Banakar *et al*., 2019), immature wheat embryos (Liang *et al*., 2017), immature maize embryos (Svitashev *et al*., 2016), soybean shoot apical meristems (Kuwabara *et al*., 2024) and *in vitro‐*fertilized rice zygotes (Toda *et al*., 2019) via PEG‐ or bombardment‐mediated transformation, and targeted mutants have been obtained after regeneration. These studies demonstrate that RNP is a practical and efficient way to obtain transgene‐free mutants in plants. However, RNPs have rarely been used to make precision genomic changes such as base editing and prime editing in plants (Zhang *et al*., 2021). The reason may be that obtaining genome editing systems in the form of purified proteins is time‐consuming and complex (Lin *et al*., 2022; Qin *et al*., 2022; Wei *et al*., 2023). *In vitro*‐transcribed (IVT) mRNA offers a simpler, more flexible and cost‐effective approach to programmed production (Lin *et al*., 2022; Qin *et al*., 2022; Wei *et al*., 2023). Our laboratory has developed an mRNA delivery system (named TECCRNA) to create CRISPR‐Cas9‐mediated gene knockouts and C‐to‐T base editing changes in wheat (Zhang *et al*., 2016, 2019), and others have reported the production of targeted mutations in lettuce protoplasts by delivering genome editing systems in the form of mRNA (Mok *et al*., 2022). Nevertheless, genome editing with current mRNA delivery systems remains inefficient and challenging in plants.

In the present study, we developed an enhanced mRNA delivery system in plants by improving the efficiency of translation of IVT mRNA and its stability during particle bombardment‐mediated transformation. We found that extending the poly(A) tail, optimizing the 5′UTR, and coating the mRNA with protamine had synergistic effects in improving editing efficiencies of gene knockouts and C‐to‐T and A‐to‐G base changes in rice and wheat. This approach outperformed the plasmid‐based transient delivery system via particle bombardment. The optimized mRNA delivery system increases the flexibility and applicability of transgene‐free genome editing in plants.

## Results

### Improving genome editing efficiency by optimizing the translation of IVT mRNA

**Figure 1:**
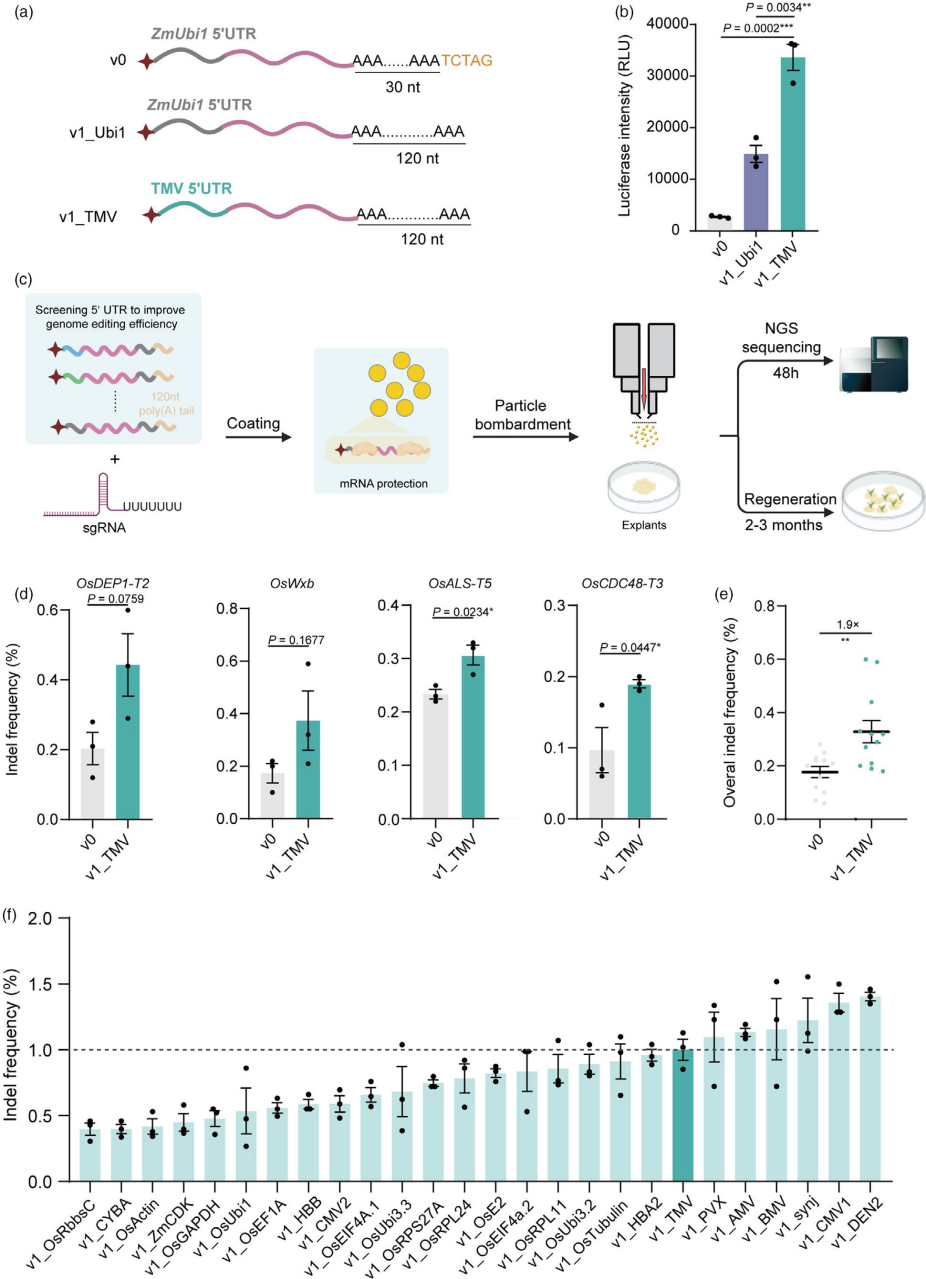
Improving genome editing efficiency by optimizing the translation of IVT mRNA. (a) Schematic representation of IVT mRNA constructs v0, v1_Ubi1(v0_120A) and v1_TMV. (b) Luciferase activities generated by v0, v1_Ubi1 and v1_TMV 18 h after transfection (*n* = 3). (c) Schematic diagram of the procedure for optimizing the particle bombardment‐mediated mRNA delivery system. The bombarded explants were collected for amplicon deep sequencing 48 h post transformation (transient system) or were used for regeneration. (d) Comparison of CRISPR‐Cas9 editing frequencies induced by v0 and v1_TMV in rice suspension cells at four target sites (*n* = 3). (e) Overall CRISPR‐Cas9 editing frequencies induced by v0 and v1_TMV in rice suspension cells (*n* = 12). (f) CRISPR‐Cas9 editing frequencies of v1 IVT mRNAs with different 5′UTRs (*n* = 3). The data were normalized to the average CRISPR‐Cas9 editing efficiencies of v1_TMV. Data in (d–f) are presented as mean ± SEM. *P* values were obtained using the two‐tailed Student's *t*‐test: *P < 0.05, **P < 0.01, ***P < 0.001.

We hypothesized that the translatability of the IVT mRNA and its stability during delivery were major limitations of current mRNA delivery systems for genome editing in plants. Since the poly(A) tail and untranslated region (UTR) have been shown to control the efficiency of translation of mRNAs (Kowalski *et al*., 2019; Pardi *et al*., 2018; Passmore and Coller, 2022), we optimized these regions, hoping to improve the translation of IVT mRNA in plant cells, and we evaluated these parameters using rice protoplasts (Figure S1a). The IVT mRNA construct (Zhang *et al*., 2016) (designated v0 this study) in our previous RNA delivery system (TECCRNA) used mRNA with a 30 nt poly(A) tail and a TCTAG overhang at the 3′ end (Figure S1a,b). To assess the influence of the poly(A) tail on protein production, we synthesized four IVT mRNA constructs with poly(A) tails of varying poly(A) lengths and no overhang, by replacing the *Xba*I restriction enzyme to *Bsa*I: this yielded variants v0_30A, v0_80A and v0_120A (Figures S1b and S2). To test these poly(A) tail variants, we synthesized IVT mRNA constructs encoding the firefly luciferase (Fluc) open reading frame (ORF) as their coding sequence (CDS) (Figures S1 and S2). Equal amounts of mRNA variants were delivered into rice protoplasts, and luciferase activity was measured at different times post‐transformation. We observed a progressive increase in luciferase activity with length of poly(A) tails ranging from 30 to 120 nucleotides (Figure S1b). v0_120A was renamed v1_Ubi1. The 5′UTR from the tobacco mosaic virus (TMV) has been widely used to increase protein expression in many transgenic vectors by inserting it into the 5′UTRs of target genes (Gallie, 2002; Gallie and Walbot, 1992). Therefore, we synthesized an mRNA construct encoding Fluc with the TMV 5′UTR and 120 nt poly(A) tail (referred to as v1_TMV hereafter) (Figure 1a; Figure S2, Table S1). We transfected rice protoplasts with v0, v1_Ubi1 and v1_TMV and found that v1_TMV produced the highest luciferase activity, about 12.9‐fold that of v0 and 2.3‐fold that of v1_Ubi1 (Figure 1b). These results demonstrated that the modification of the IVT mRNA construct, v1_TMV, using the TMV 5′UTR and a 120 nt poly(A) tail significantly increases protein yield in rice protoplasts. To test whether v1_TMV could be used to improve genome editing, we applied it in genome editing using particle bombardment (Figure 1c), as this method has been demonstrated to induce edited plants in a wide range of species (Altpeter *et al*., 2016; Gao, 2021). We prepared v1_TMV and v0 expressing Cas9 (Figure S3) and delivered them along with sgRNA targeting four endogenous sites (Figure 1d) into suspension cells of rice variety Zhonghua 11 by particle bombardment. Forty‐eight hours after transformation, we isolated genomic DNA for deep amplicon sequencing (Figure 1c). We found that the efficiency of edits induced by v1_TMV was higher than that of v0 at all four target sites, and the average editing frequency of v1_TMV was 1.9‐fold higher than that of v0 (Figure 1d, e). Taken together, these results demonstrate that the editing efficiency of v1_TMV is higher than that of v0 when using particle bombardment in rice suspension cells.

5′UTR have been shown to control the efficiency of translation of IVT mRNAs (Asrani *et al*., 2018; Leppek *et al*., 2022). To further enhance genome editing efficiency, we used the transient particle bombardment‐mediated mRNA delivery system to examine the effects of a variety of 5′UTR sequences in rice suspension cells (Figure 1c). These 5′UTR sequences were derived from constitutively expressed genes of both plants and animals as well as from viruses (Table S1). We identified several 5′UTR sequences with comparable or better performance in CRISPR‐Cas9 editing than TMV at *OsDEP1‐T2* (Table S2), among which 5′UTR DEN2 from dengue virus exhibited the highest editing efficiency (Figure 1f). We named this mRNA construct v1_DEN2 (Figure S3).

### Optimizing particle bombardment‐mediated mRNA delivery to suspension cells and immature embryos

**Figure 2:**
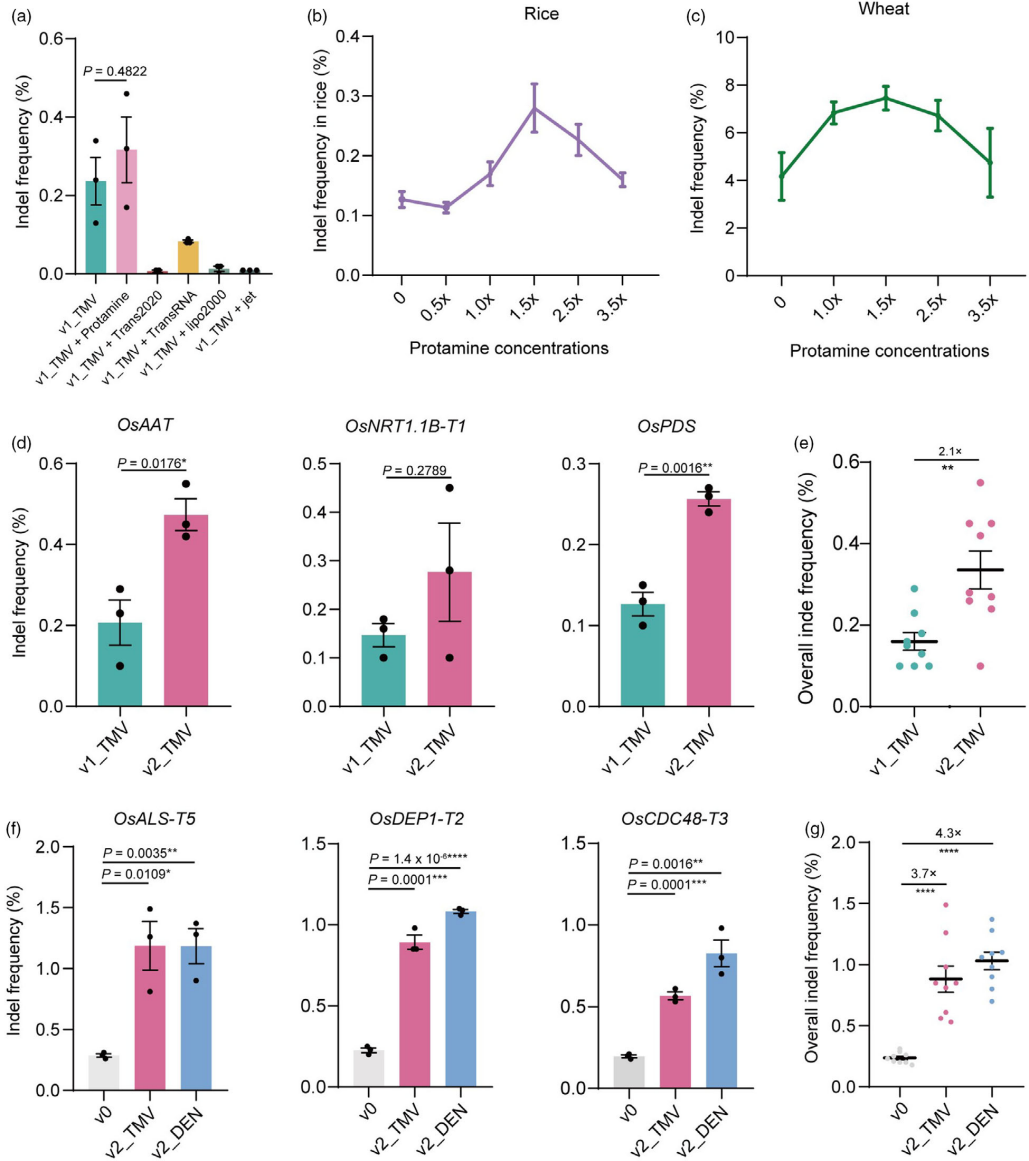
Optimizing particle bombardment‐mediated mRNA delivery. (a) CRISPR‐Cas9 editing frequencies of v1_TMV coating with protamine, TransIT‐2020, TransIT‐mRNA, Lipofectamine 2000 and jetPRIME (*n* = 3). (b) CRISPR‐Cas9 editing efficiencies of v1_TMV with different protamine concentrations in rice suspension cells (*n* = 3). (c) CRISPR‐Cas9 editing efficiencies of v1_TMV with different protamine concentrations in wheat immature embryos (*n* = 3). (d) Comparison of CRISPR‐Cas9 editing efficiencies of v1_TMV and v2_TMV at three more target sites in rice suspension cells (*n* = 3). (e) Overall editing frequencies of v1_TMV and v2_TMV in rice suspension cells (*n* = 9). (f) Comparative analysis of CRISPR‐Cas9 editing efficiencies induced by v0, v2_TMV and v2_DEN2 at three target sites in rice suspension cells (*n* = 3). (g) Overall editing frequencies of CRISPR‐Cas9 using v0, v2_TMV and v2_DEN2 in rice suspension cells (*n* = 9). All data are presented as mean ± SEM. *P* values were obtained using the two‐tailed Student's *t*‐test: *P < 0.05, **P < 0.01, ***P < 0.001, ****P < 0.0001.

Given that RNA is an intrinsically unstable molecule and that RNA molecules are naked in our basic particle bombardment delivery system, they could be prone to degradation. Therefore, we hypothesized that coating the RNA with a protecting agent might further improve editing efficiency. Coating mRNA with agents, such as cationic lipids, polymers and cationic peptides, is known to protect it from degradation during delivery in many mammalian cell lines (Jarzebska *et al*., 2021; Pardi *et al*., 2018). So, we compared the effects of five commercially available reagents, TransIT‐2020, TransIT‐mRNA, Lipofectamine 2000, jetPRIME and protamine on delivery of mRNA to plant cells by particle bombardment. We prepared v1_TMV expressing Cas9 (Figure S3) and the sgRNA targeting rice *OsDEP1‐T2* (Table S2) with each of these five agents separately before delivering them into rice suspension cells by particle bombardment (Figure 1c). Amplicon sequencing showed that coating mRNA with protamine led to higher editing efficiency than naked mRNA, and the four other methods were less effective (Figure 2a). Protamine and RNA were mixed in a 1:1 mass ratio in our initial experiment, and we decided to compare the following mass ratios, 2:1, 1:1, 1:1.5, 1:2.5 and 1:3.5 (represents 0.5×, 1×, 1.5×, 2.5× and 3.5× protamine concentration) in rice suspension cells. The 1:1.5 mass ratio had the highest editing efficiency (Figure 2b). To see whether coating mRNA with protamine was also useful in other plants, we tested four mixing ratios (1:1, 1:1.5, 1:2.5 and 1:3.5) in wheat immature embryos 48 post particle bombardment (Figure 1c). The 1:1.5 mass ratio turned out to be also optimal in wheat (Figure 2c). We named the mRNA delivery system protecting the improved IVT mRNA constructs v1_TMV/DEN2 with protamine at the 1:1.5 ratio v2_TMV/DEN2. We then tested v2_TMV using CRISPR‐Cas9 mRNA at three more sites in rice suspension cells (Figure 2d; Table S2). v2_TMV gave higher editing rates at all three targets and was overall 2.1‐fold more effective than naked mRNA in rice suspension cells (Figure 2d,e). Compared with v0, v2_TMV and v2_DEN2 had about 3.7‐fold and 4.1‐fold higher editing efficiencies, respectively, at three target sites in rice suspension cells (Figure 2f,g; Table S2). Overall, these results show that coating the mRNA with protamine at a 1:1.5 ratio further improves the efficiency of mRNA delivery in rice and wheat.

### The mRNA‐based v2_TMV/DEN2 system has enhanced editing activity compared with the plasmid‐based system (TECCDNA) in suspension cells and immature embryos

**Figure 3:**
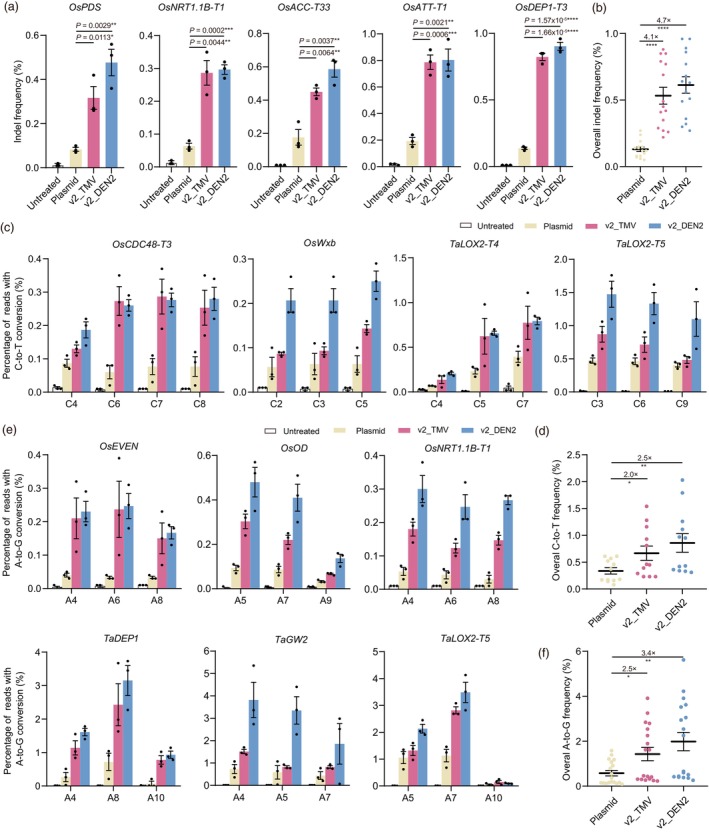
Comparison of editing frequencies using v2_TMV/DEN2 and the plasmid‐based system (TECCDNA) in suspension cells and immature embryos. (a) Comparison of CRISPR‐Cas9 editing efficiencies of plasmid, v2_TMV and v2_DEN2 in rice suspension cells at five target sites (*n* = 3). (b) Overall CRISPR‐Cas9 editing frequencies of plasmid, v2_TMV and v2_DEN2 in rice suspension cells (*n* = 15). (c) Comparison of single C‐to‐T conversion frequencies of plasmid, v2_TMV and v2_DEN2 in rice suspension cells and wheat immature embryos at four target sites (*n* = 3). (d) Overall C‐to‐T conversion (*n* = 12) frequencies of plasmid, v2_TMV and v2_DEN2. (e) Comparison of single A‐to‐G conversion frequencies of plasmid, v2_TMV and v2_DEN2 in rice suspension cells and wheat immature embryos at six target sites (*n* = 3). (f) Overall A‐to‐G conversion (*n* = 18) frequencies of plasmid, v2_TMV and v2_DEN2. All data are presented as mean ± SEM. *P* values were obtained using the two‐tailed Student's *t*‐test: *P < 0.05, **P < 0.01, ***P < 0.001, ****P < 0.0001.

The plasmid‐based transient delivery system has been proven effective in generating transgene‐free mutants in plants (Hamada *et al*., 2018; Hoengenaert *et al*., 2023; Li *et al*., 2022; Qiu *et al*., 2022; Zhang *et al*., 2019). In 2017, our lab developed a bombardment‐mediated DNA transient delivery system, TECCDNA, and applied it to transgene‐free genome editing in wheat (Zhang *et al*., 2016). TECCDNA had a higher editing efficiency than the RNA delivery system (TECCRNA) (Zhang *et al*., 2016). Given that v2_TMV and v2_DEN had improved editing efficiencies, we compared their performance with TECCDNA in CRISPR‐Cas9, C‐to‐T and A‐to‐G base editing (Figure S3) in rice and wheat. For CRISPR‐Cas9 editing, we evaluated five targets (Table S2) in rice suspension cells. As expected, v2_TMV and v2_DEN2 had significantly higher editing efficiencies, with v2_DEN yielding the highest editing rates at all target sites (Figure 3a). v2_TMV and v2_DEN2 gave on average 4.1‐ and 4.7‐fold improvements, respectively, over the plasmid (Figure 3b). For C‐to‐T and A‐to‐G base transversions, we chose four sites for C‐to‐T editing (Table S2), two from rice and two from wheat (Figure 3c,d) and six sites (Table S2) for A‐to‐G editing, three from rice and three from wheat (Figure 3e,f). v2_TMV and v2_DEN2 exhibited higher base editing efficiencies than the plasmid at all these targets for both C‐to‐T and A‐to‐G base changes and v2_DEN2 had the highest efficiency at most of the targets (Figure 3c,e). The editing efficiencies of v2_DEN2 were an average 2.5‐ and 3.4‐fold higher than plasmid for C‐to‐T and A‐to‐G base changes, respectively (Figure 3d,f). Collectively, these results demonstrate that we have established an efficient particle bombardment‐mediated mRNA delivery system (v2_TMV/DEN2) that is more efficient than plasmid‐based delivery.

### v2_TMV/DEN2 perform edits in rice and wheat

**Figure 4:**
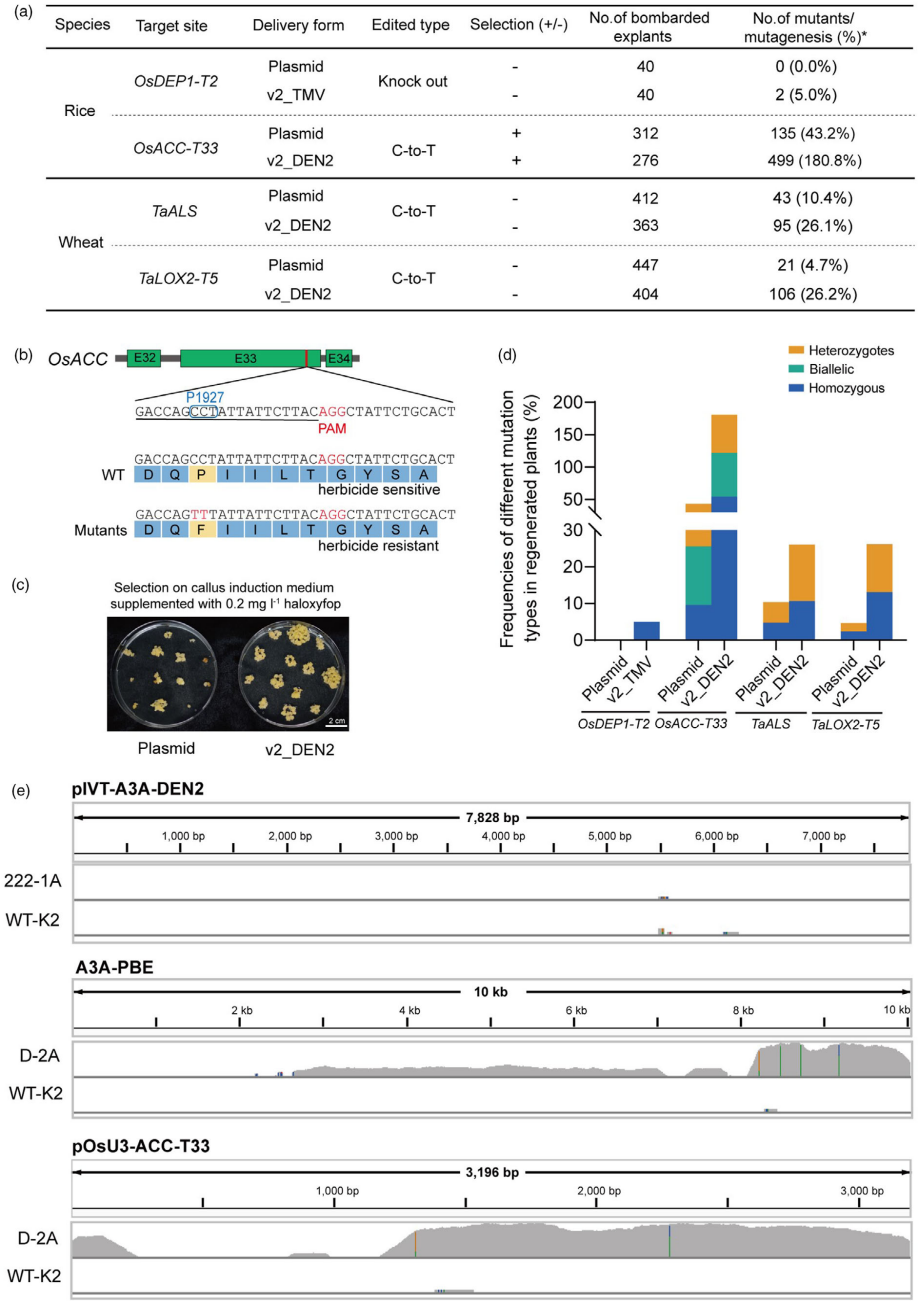
Editing of plants with the enhanced mRNA delivery systems v2_TMV and v2_DEN2. (a) Comparison of editing frequencies achieved by delivering genome editing systems with plasmid or v2_TMV or v2_DEN2 in regenerated rice or wheat plants. *Based on the number of plants carrying the observed mutations over the total number of bombarded explants. (b) *OsACC‐T33* with the P1927F substitution confers resistance to herbicides. Sequence alignment comparing WT *OsACC‐T33* with that of the T_0_ mutant. The protospacer‐adjacent motif (PAM) is highlighted in red, and the protospacer is underlined. (c) Phenotypes of WT (left) and *OsACC* P1927F mutants (right) grown in medium supplemented with 0.2 mg/L haloxyfop. (d) Frequencies of the different mutation types among T_0_ mutants. (e) Whole‐genome sequencing (WGS) was performed on T0 mutants generated using v2_DEN2 (222‐1A) or transformed with A3A‐PBE and pOsU3‐ACC‐T33 (D‐2A). Wild‐type Kitaake (WT‐K2) was used as a negative control.

To see whether v2_TMV/DEN2 was effective at the plant level, we chose two target sites each in rice and wheat and compared v2_TMV/DEN2 with plasmid‐based delivery. Regenerated plants were identified by polymerase chain reaction‐restriction enzyme (PCR‐RE) or amplicon sequencing. We found that v2_TMV generated 5.0% knockout edits compared to 0% induced by plasmid at the *OsDEP1‐T2* target site (Figure 4a; Figure S4a). It has previously been shown that two C‐to‐T base conversions (P1927F) in rice acetyl‐coenzyme A carboxylase (*OsACC*) endow rice with resistance to haloxyfop herbicide (Li *et al*., 2020) (Figure 4b). So, we transformed calli with plasmid and v2_DEN2 targeting *OsACC‐T33* (Table S2) and selected for haloxyfop resistance during subsequent tissue culture. Forty days after transformation, we found that v2_DEN2 produced more calli with vigorous growth than plasmid (Figure 4c). Resistant calli were transferred to a regeneration medium to obtain regenerated plants. Examination of these lines by amplicon sequencing revealed a mutation efficiency of 180.8% induced by v2_DEN2 (all shoots regenerated from explants were sampled, and critically, some explants formed more than one shoot. For transformation by v2_DEN2, 276 explants were bombarded and we obtained 499 mutants, yielding an efficiency of 180.8%), which was 4.2‐fold higher than that induced by plasmid (43.2%) (Figure 4a,d; Figure S4b,c). The 499 mutants induced by v2_DEN2 consisted of 162 (58.7%, 162/276) biallelic mutants, 188 (68.1%, 188/276) heterozygous mutants and 149 (54.0%, 149/276) homozygous mutants (Figure 4d).

We also transformed wheat immature embryos with v2_DEN2 and plasmid and found that v2_DEN2 increased the C‐to‐T editing efficiency to 26.1% compared to the 10.4% induced by plasmid at the *TaALS* (Figure 4a; Figure S5a,b, Table S2); it also improved C‐to‐T editing efficiency to 26.2% compared to 4.7% at *TaLOX2‐T5* (Figure 4a; Figure S5c,d, Table S2). The frequencies of homozygous mutants were increased from 4.8% (20/412) and 2.4% (11/447) to 10.7% (39/363) and 13.1% (53/404) at the *TaALS* and *TaLOX2‐T5* target sites, respectively (Figure 4d). Overall, v2_TMV/DEN2 yielded 4.3‐fold and 3.5‐fold improvements in editing efficiencies compared with plasmid‐based delivery systems across rice and wheat.

To confirm the absence of genomic insertion of exogenous fragments with our improved mRNA delivery system, we chose the two mutants obtained with v2_TMV encoding Cas9 and randomly chose four mutants generated with v2_DEN2 encoding A3A‐PBE, six transformed with a plasmid encoding A3A‐PBE by biolistic delivery and four wild‐type rice plants (one ZH11 and three Kitaake) as control and performed whole‐genome sequencing (Figure S6a). We detected the presence of exogenous fragments insertion (about 10 000–30 000 reads mapped to plasmids (A3A‐PBE and pOsU3‐ACC‐T33)) in two mutants (D‐2A and D‐4A) out of six derives obtained by plasmid‐based delivery. In contrast, we found no exogenous fragments in any of the six sequenced mutants (643, 651, 222‐1A, 222‐2A, 222‐3A and 222‐4A) obtained with our improved mRNA delivery system v2_TMV/DEN2. We noticed a few reads (1–20) that mapped to the IVT construct (pIVT‐Cas9‐TMV or pIVT‐A3A‐DEN2) in the mutants obtained by mRNA‐based delivery, and we speculate that they were the result of aerosol pollution or sequencing error because we also found a few (1–20) reads that mapped to plasmids (A3A‐PBE and pOsU3‐ACC‐T33) and IVT construct (pIVT‐Cas9‐TMV or pIVT‐A3A‐DEN2) among the reads of wild‐type plants (Figure 4e; Figure S6b). These results confirmed that our improved mRNA delivery system outperforms plasmid‐based delivery and is effective in producing transgene‐free derivatives in the T_0_ generation.

## Discussion

Through extending the poly(A) tail, optimizing the 5′UTR and protecting the mRNA with protamine, we have developed an improved particle bombardment‐mediated mRNA delivery system named v2_TMV/DEN2. The system showed higher genome editing activity than the original v0 system (TECCRNA (Zhang *et al*., 2016)). Importantly, v2_TMV/DEN2 was more effective in producing knockouts, and A‐to‐G and C‐to‐T edits than the plasmid‐based transient delivery system (TECCDNA) in rice suspension cells and wheat immature embryos. In addition, v2_TMV/DEN2 generated transgene‐free genome‐edited plants in the T_0_ generation.

Transgene‐free genome editing technology is valuable for reducing public concerns regarding the environmental and food safety of gene‐edited products and so is crucial for the advancement of gene‐edited crop breeding (Li *et al*., 2024). Achieving transgene‐free genome editing in the T_0_ generation is highly desirable as it eliminates the need for genetic segregating out transgenes by backcrossing or selfing, which is often labour‐intensive and time‐consuming (Gao, 2021). This is particularly relevant for clonally propagated crops like certain rice varieties where transgene removal is not feasible (Wang *et al*., 2019).

Currently, *Agrobacterium tumefaciens*‐mediated transformation and particle bombardment are the two widely used methods for delivering genome editing components into plant cells (Altpeter *et al*., 2016). Both T‐DNA (*Agrobacterium tumefaciens* infection) and plasmid DNA (particle bombardment) can be transiently expressed in nuclei without being integrated into the host chromosome and so can generate transgene‐free T_0_ plants (Huang *et al*., 2023; Zhang *et al*., 2016). However, in both methods, the possible integration of small DNA fragments into the plant genome poses challenges for detection by PCR. Thus, DNA‐independent delivery of editing reagents, such as RNP complexes or IVT mRNA, can mitigate these risks. Owing to its programmability, scalability and ease of design and synthesis, RNA‐based delivery has pivotal applications in protein therapy, vaccination and gene editing (Chen *et al*., 2024; Kowalski *et al*., 2019). In plants, delivery of genome editing components in the form of RNA by transfecting lettuce protoplasts (Toda *et al*., 2019) and wheat embryos (Zhang *et al*., 2016) has also been demonstrated. However, plant regeneration from protoplasts is technically difficult and highly inefficient for many monocot crop species. Therefore, we have developed a complementary efficient mRNA delivery system v2_TMV/DEN2.

In 2017, our lab described the development of particle bombardment‐mediated RNA delivery system (TECCRNA) and applied it to transgene‐free genome editing in wheat (Zhang *et al*., 2016). However, the mutagenesis frequency with TECCRNA was lower than that obtained with plasmid‐based transient delivery system (TECCDNA) in a side‐by‐side experiment (Zhang *et al*., 2016), limiting its effectiveness for transgene‐free genome editing. Low translation ability, poor mRNA stability and low delivery efficiency are primary constraints of mRNA‐based delivery systems in plants. To improve the translatability of IVT mRNA, we optimized the main regulatory regions in the IVT mRNA including poly(A) tail and 5′UTR. The optimized IVT mRNA construct with the TMV 5′UTR and a 120 nt poly(A) tail significantly increased protein yield compared with the mRNA construct v0 in TECCRNA (Figure 1b). To increase the stability of the mRNA during bombardment and optimize the bombardment‐mediated delivery system, we coat the mRNA with several agents which are known to protect mRNA from degradation during delivery in many mammalian cell lines (Jarzebska *et al*., 2021; Pardi *et al*., 2018). It showed that coating the mRNA with protamine had the best effect (Figure 2a). This result suggested a distinct difference in delivery mechanisms between plant and mammalian cells and supports our conclusion on the need for further optimization of mRNA delivery systems tailored for plants. In addition, the chemically synthesized sgRNAs (Hendel *et al*., 2015) used in this work are known to be more stable. The plasmid‐based DNA delivery system used, TECCDNA, has previously been demonstrated to produce plants with highly targeted mutation frequencies (Hamada *et al*., 2018; Hoengenaert *et al*., 2023; Li *et al*., 2022; Qiu *et al*., 2022; Zhang *et al*., 2019). Our v2_TMV/DEN2 mRNA outperformed TECCDNA, confirming its utility for transgene‐free genome editing. The full‐fledged commercial kits used for IVT mRNA preparation, and modified sgRNAs bought from a biotechnology company, help to overcome the technical barriers to the practical application of our improved mRNA delivery system. The higher editing efficiency should also reduce the workload involved in transformation, tissue culture and mutant identification and so compensate for the costs of mRNA and sgRNA preparation. We therefore believe that our improved mRNA delivery system will be useful for genome editing by bombardment‐mediated delivery and will make it much easier to obtain transgene‐free derivatives in T_0_.

Liu and colleagues have shown that biolistic transformation can result in genomic damage in transgenic rice and maize. This damage includes chromosome truncations, large deletions and other damage (Liu *et al*., 2019). We think the recent advances in bio‐macromolecule delivery using nanotechnology may help to address this problem. Studies have shown that layered double hydroxide nanoparticles and carbon nanotubes are able to deliver siRNA into intact plant cells by spraying or injection without the destruction of plant tissue (Demirer *et al*., 2020; Jain *et al*., 2022). Therefore, in future, the use of nanoparticles may replace the use of particle bombardment for mRNA delivery.

In conclusion, we have established an efficient mRNA delivery system v2_TMV/DEN2 and obtained transgene‐free mutants in rice and wheat in the T_0_ generation at high frequency. Our strategy, based on bombardment‐mediated delivery of CRISPR reagents into plant tissues, has the potential to be applied to other plant species amenable to biolistic delivery, including major crops like maize, sorghum, soybean, barley and vegetative propagated crops, such as sugarcane and banana, where transgene segregation through backcrossing or selfing is challenging. We believe that this improved mRNA delivery system will accelerate advances in plant genome editing and provide a useful tool for plant genetic improvement.

## Experimental procedures

### Plasmid construction

To construct pIVT‐LUC‐30A, pIVT‐LUC‐80A and pIVT‐LUC‐120A vectors (Figure S2) used for *in vitro* transcription of v0_30A, v0_80A and v0_120A, respectively, we synthesized 30, 80 and 120 nt poly(A) tails flanked by *Bsa*I restriction enzyme sites and replaced the 30 nt poly(A) tail flanked by an *Xba*I restriction enzyme site in pLZT7 (Liang *et al*., 2017) (v0). We then cloned the CDS of *Fluc* and inserted it between the *Kpn*I and *Hin*dIII restriction enzyme sites, replacing the CDS of *Cas9* in v0, v0_30A, v0_80A and v0_120A (v1_Ubi1) to construct plasmids for *in vitro* transcription of *Fluc*. To construct vector pIVT‐Cas9‐TMV (Figure S2), we replaced the CDS of *Fluc* with the CDS of *Cas9*. To construct vectors used for IVT mRNAs with different 5′UTRs and 120 nt poly(A) tail, we synthesized the different 5′UTR sequences listed in Table S1 and replaced the 5′UTR of TMV in pIVT‐Cas9‐TMV. To construct the plasmids used for *in vitro* transcription of cytidine base editor (CBE) and adenine base editor (ABE), we cloned the CDSs from plasmids pH‐ABE8e (Xue *et al*., 2023) and A3A‐PBE (Zong *et al*., 2018) and used them to replace the CDSs of Cas9 in pIVT‐Cas9‐TMV and pIVT‐Cas9‐DEN2 (Figure S3).

### Preparation of mRNA and sgRNA

Preparation of mRNAs followed *in vitro* transcription from DNA templates. The latter were linearized plasmids that were digested with *Xba*I or *Bsa*I (NEB, Ipswich, England) and purified using a Thermo Scientific GeneJET Gel Extraction Kit (K0692). *In vitro* transcription was performed with a HiScribe™ T7 High Yield RNA Synthesis Kit (E2040S; NEB, Ipswich, England). Twenty microlitres of transcription reaction contained 1 μg linear DNA template, 10 mM of each NTP, 2 μL T7 RNA polymerase mix and 1× transcription buffer. After incubation for 2 h at 37 °C, the DNA was digested by the addition of 1 μL DNase I (M0303S; NEB, Ipswich, England) for 15 min at 37 °C. Then it was purified with a Monarch RNA Cleanup Kit (T2040; NEB, Ipswich, England). mRNA concentrations were determined on a Nanodrop 2000 (Thermo Fisher Scientific, Waltham, USA). *In vitro* transcribed mRNA was then m7G‐capped using the Vaccinia Capping System (M2080S; NEB, Ipswich, England). The sgRNAs used in the experiment were chemically synthesized and purchased from GenScipt (Nanjing, China).

### Transfection of rice protoplasts

The *Japonica* rice variety Zhonghua 11 was used to generate protoplasts. About 14‐day‐old rice seedlings cultured at 27 °C on MS medium with a 16‐h light/8‐h dark cycle were used for protoplast isolation. Protoplast isolation was performed as previously described (Shan *et al*., 2014). Samples of 5 μg of mRNA were used for PEG‐mediated transfections with the Fluc reporter. After incubation, protoplasts were collected for extraction of protein, and Fluc activity was measured using the Luciferase Reporter Assay System (Promega, Madison, USA).

### Biolistic delivery of plasmid and RNA and plant regeneration

Cas9, CBE or ABE and sgRNA in the form of plasmid DNA or RNA were used to bombard rice suspension cells. sgRNAs were purchased from GenScript. DNA were delivered via particle bombardment as previously described (Liang *et al*., 2018; Shan *et al*., 2014). For standard RNA delivery, experiments were performed as follows: 10 μg mRNA (20 μL) and 10 μg sgRNA were mixed gently and thoroughly with 50 μL gold particles (40 mg/mL; Bio‐Rad, California, USA) for 10 shots. Ten microlitres of ammonium acetate (5 m) and 200 μL of 2‐propanol were then added and mixed thoroughly in turn to precipitate the mRNA and sgRNA onto gold particles. After incubation at −20 °C for 2 h, the mixture was centrifuged at 10 000 ***g*** for 10 s at room temperature, and the supernatant was discarded. The pellet was resuspended in 1 mL absolute ethanol, centrifuged at 10 000 ***g*** for 10 s at room temperature and discard the supernatant, and then resuspend the pellet carefully in 200 μL absolute ethanol. It was then loaded onto a microcarrier (20 μL for each shot) by pipette and allowed to air dry.

For RNA delivery with protamine (P4020; Sigma‐Aldrich, Milwaukee, WI, USA), experiments were performed as follows: for 10 shots, 10 μg mRNA (20 μL) and 10 μg sgRNA (10 μL) were mixed gently and thoroughly with 50 μL gold particles (40 mg/mL, Bio‐Rad, California, USA). Approximately 6.7, 13.4, 20, 33.5 or 46.9 μL 1 mg/mL protamine water solution (0.5×, 1×, 1.5×, 2.5× and 3.5× concentrations respectively) were then added and mixed thoroughly. After incubation on ice for 10 min, 10 μL ammonium acetate (5 m) and 200 μL 2‐propanol were added and mixed thoroughly in turn to precipitate mRNA or sgRNA onto the gold particles. The remaining steps were performed as described in the previous work (Liang *et al*., 2018).

Rice suspension cells were spread as thinly as possible on the medium in a circle about 1 cm in diameter and particle bombardments were performed using a PDS‐1000/He system with a target distance of 6.0 cm and helium pressure of 1350 psi. Particle bombardment and tissue culture of rice calli and wheat immature embryos were performed as previously described (Shan *et al*., 2014; Zhang *et al*., 2016). About 0.2 mg/L haloxyfop was used for the selection of resistant rice calli (Li *et al*., 2020).

### DNA extraction

We used 2× CTAB solution to extract the genomic DNA of rice suspension cells, wheat immature embryos and leaves from regenerated plants. It was quantified with a NanoDrop 2000 spectrophotometer (Thermo Fisher Scientific, Waltham, USA).

### Next‐generation sequencing

We designed two rounds of PCR amplification. In the first round, we amplified target site sequences from genomic DNA with site‐specific primers. In the second, amplification primers containing forward and reverse barcodes were added to the PCR products for library construction. The amplified products were purified using a Thermo Scientific GeneJET Gel Extraction Kit (K0692) and quantified with a NanoDrop 2000 spectrophotometer (Thermo Fisher Scientific, Waltham, USA). Equal amounts of PCR product were pooled and sequenced commercially (Novogene, Tianjing, China) using a NovaSeq platform. Sequencing of each amplicon was carried out three times, using genomic DNA from three independent samples. Primers are listed in Table S3.

### Mutant identification by next‐generation sequencing, Sanger sequencing and PCR‐RE

Many plantlets were regenerated in the absence of herbicide selection in our assays. We combined the plantlets in pools (each pool usually contained three to four plantlets) to detect the mutations by next‐generation sequencing. Plantlets in the pools that gave positive signals were tested one by one by Sanger sequencing. T_0_ rice plants regenerated with herbicide selection were examined individually by Sanger sequencing. Primers are listed in Table S3.

Regenerated wheat lines were identified by PCR‐RE assays. These were performed to identify wheat mutants with C‐to‐T conversions in target regions, as described previously (Qiu *et al*., 2022). For wheat, plantlets (usually three to four plantlets) were pooled for the assays, and the positive pools were examined further to identify individual mutant plantlets. Primers are listed in Table S3.

### Analysis of whole‐genome sequencing data

A total of 18 plants, including two edited plants (T0‐643 and T0‐651) and two wild‐type plants regenerated after delivery of v2_TMV encoding Cas9 delivery (T0‐641 and T0‐653), one ZH11 wild‐type plant, four mutants generated with v2_DEN2 encoding A3A‐PBE, six mutants transformed by a plasmid encoding A3A‐PBE via biolistic delivery and three Kitaake wild‐type rice plant were used to test for the presence of exogenous fragments. They were sequenced with a NovaSeq platform (Novogene, Tianjing, China), and BGI generated an average of 24.03 Gb of data (~62.98×) per plant. All sequence data reads were mapped to the reference genome (*O. sativa* Kitaake_499_v3.0 and ZH11 genome: https://www.mbkbase.org/ZH11/) and DNA sequences of IVT mRNAs and sgRNA by bwa. Background was filtered by comparison with a wild‐type control to identify the positions of all insertions. The position where a sequence aligned to the reference genome identified the insertion site, and the positions where the sequence aligned to the vector identified the start and end positions of the vector.

### Statistical analysis

GraphPad Prism 8 and Microsoft Excel 2016 software were used to analyze the data. All numerical values are presented as mean ± SEM. Differences between control and treatments were tested by two‐tailed Student's *t*‐test.

## Conflict of interest

The authors declare that they have no competing interests.

## Author contributions

F.Q. and C.G. designed the project; F.Q. and C.X. performed the experiments. B.L. and J.L. performed rice and wheat transformation. Q.G. performed the whole‐genome sequencing data analysis. F.Q., C.X., B.L., R.L., K.C. and C.G. wrote the manuscript. C.G. supervised the project. All authors reviewed the manuscript.

## Ethical approval

This article does not contain any studies with human participants or animals performed by any of the authors.

## Supporting information


**Figure S1** Improving mRNA translation in protoplasts by optimizing mRNA poly(A) tail.
**Figure S2** Schematic representation of pLZT7, pIVT‐LUC‐30A, pIVT‐LUC‐80A, pIVT‐LUC‐120A and pIVT‐LUC‐TMV vectors.
**Figure S3** Schematic representation of vectors used for *in vitro* transcription of Cas9, CBE and ABE.
**Figure S4** Genotypes of genome‐edited rice mutants.
**Figure S5** The outcome of PCR‐RE assays for representative wheat mutants.
**Figure S6** The description of the whole genome sequencing data (a) and the analysis of exogenous DNA fragment insertion in mutants obtained via plasmid‐ or mRNA‐based delivery system (b).
**Table S1** Sequences of 5′UTRs used in this study.
**Table S2** Description of sgRNA sites and sequences.
**Table S3** PCR primers used in this study.

## Data Availability

All data that support the findings of this study are available in the article and supplementary information or are available from the corresponding author upon request. Sequence data are present in the Phytozome databases (https://phytozome‐next.jgi.doe.gov/). The deep sequencing data will be deposited in a National Center for Biotechnology Information (NCBI) BioProject database (accession code PRJNA1198388). Plasmids in this work will be available through Addgene.
