# Supplementary material for: An efficient mRNA delivery system for genome editing in plants
Source: Plant Biotechnol J. 2025 Feb 10;23(4):1348–58. doi: 10.1111/pbi.14591 (PMC11933840; doi:10.1111/pbi.14591)
Supplement: Supplementary file 1 — Figure S1 Improving mRNA translation in protoplasts by optimizing mRNA poly(A) tail. Figure S2 Schematic representation of pLZT7, pIVT‐LUC‐30A, pIVT‐LUC‐80A, pIVT‐LUC‐120A and pIVT‐LUC‐TMV vectors. Figure S3 Schematic representation of vectors used for in vitro transcription of Cas9, CBE and ABE. Figure S4 Genotypes of genome‐edited rice mutants. Figure S5 The outcome of PCR‐RE assays for representative wheat mutants. Figure S6 The description of the whole genome sequencing data (a) and the analysis of exogenous DNA fragment insertion in mutants obtained via plasmid‐ or mRNA‐based delivery system (b). Table S1 Sequences of 5′UTRs used in this study. Table S2 Description of sgRNA sites and sequences. Table S3 PCR primers used in this study. [file PBI-23-1348-s001.docx]

**Supplementary figures**

**
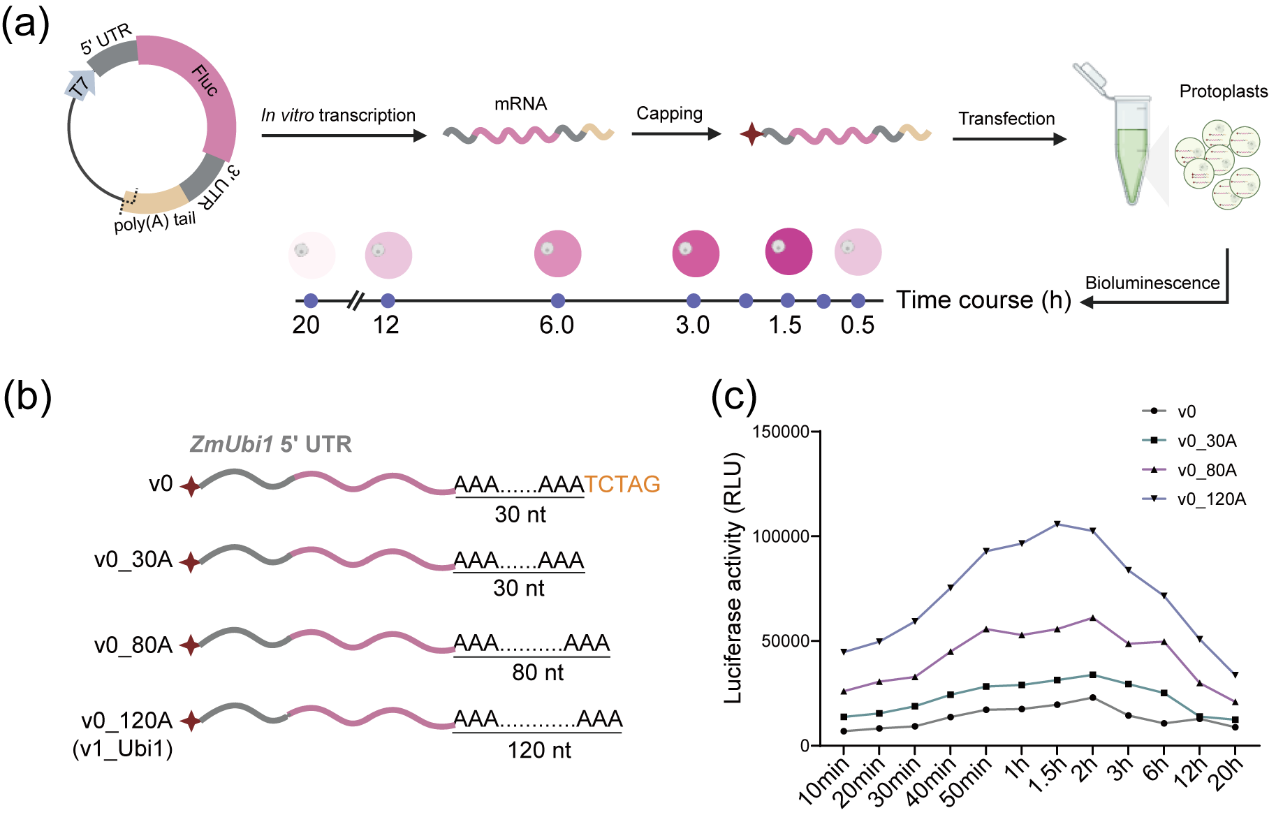
**

**Figure S1** Improving mRNA translation in protoplasts by optimizing mRNA poly(A) tail.(a) Schematic diagram of test of IVT mRNA for translatability by luciferase assay over a 20h time course after transfection of rice protoplasts. (b) Schematic representation of IVT mRNA constructs v0, v0_30A, v0_80A, v0_120A (v1_Ubi1). (c) Luciferase activities generated by IVT mRNAs with varying poly(A) lengths and with or without TCTAG overhangs over 20h time course after transfection in rice protoplasts. RLU, relative light unit.


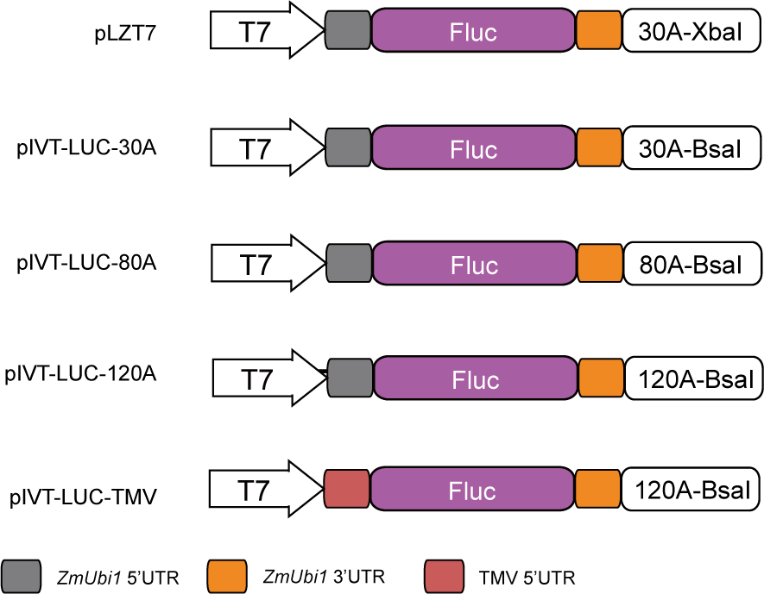


**Figure S2** Schematic representation of pLZT7, pIVT-LUC-30A, pIVT-LUC-80A, pIVT-LUC-120A and pIVT-LUC-TMV vectors.


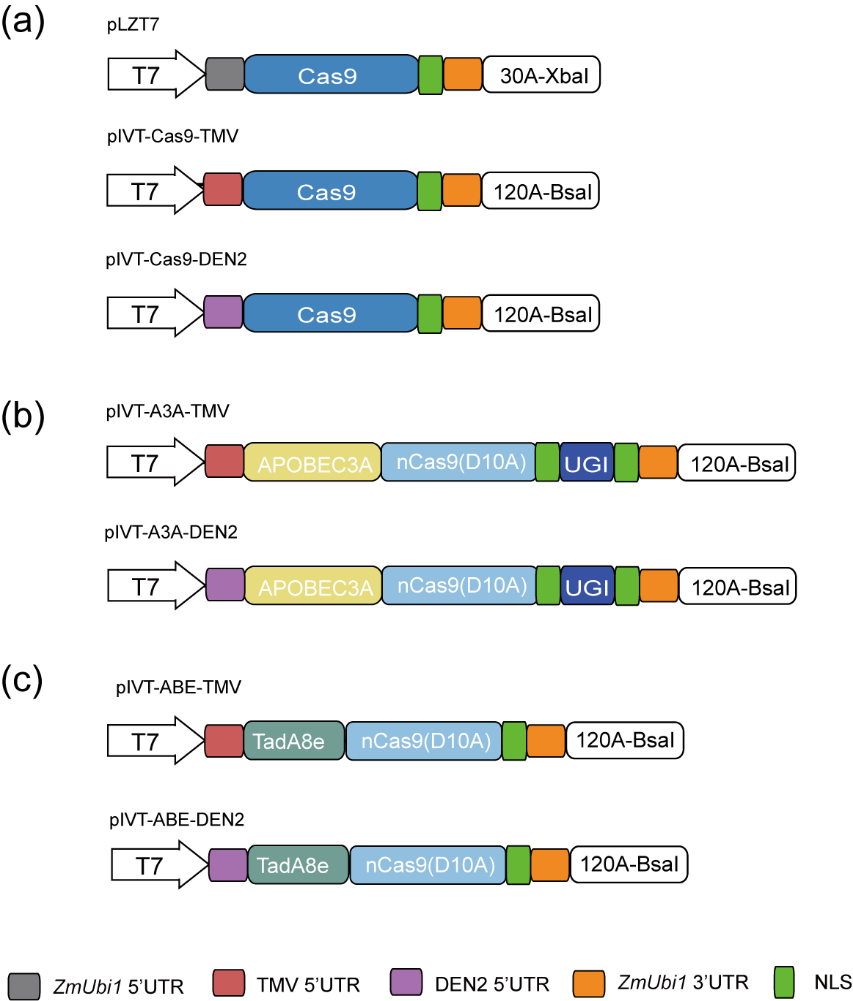


**Figure S3** Schematic representation of vectors used for *in vitro* transcription of Cas9, CBE and ABE. (a) Schematic representation of pLZT7, pIVT-Cas9-TMV and pIVT-Cas9-DEN2 used for *in vitro* transcription of Cas9 as v0, v1_TMV and v1_DEN2. (b) Schematic representation of pIVT-A3A-TMV and pIVT-A3A-DEN2 used for *in vitro* transcription of A3A-PBE as v1_TMV and v1_DEN2. (C) Schematic representation of pIVT- ABE8e -TMV and pIVT- ABE8e -DEN2 used for *in vitro* transcription of ABE8e as v1_TMV and v1_DEN2.


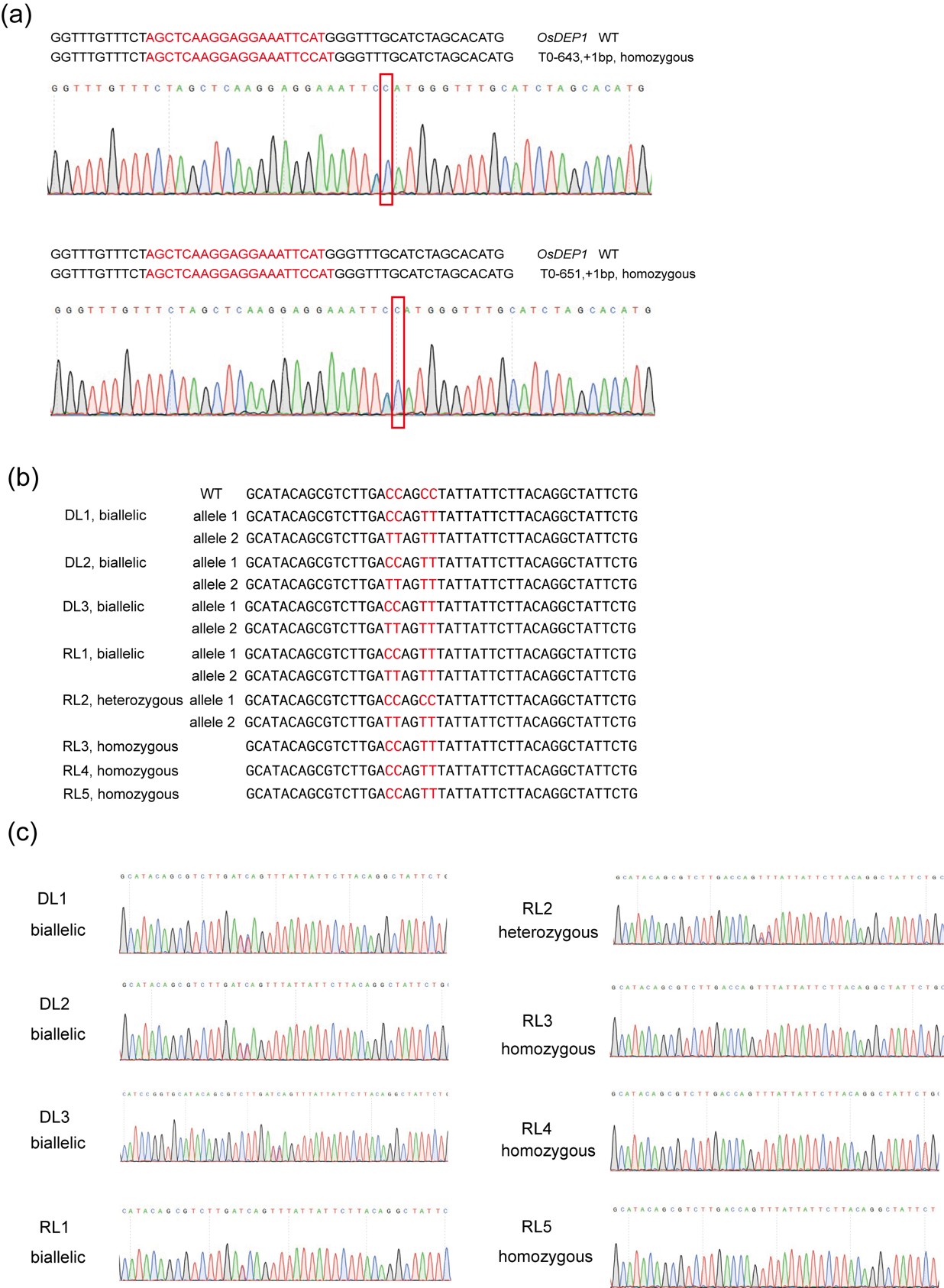


**Figure S4** Genotypes of genome-edited rice mutants. (a) Sanger sequencing chromatograms of two Cas9-edited mutants induced by v2_TMV. Red squares indicate the one base insertion edits at *OsDEP1-T2* target. (b) Genotypes of six representative rice mutants (DL1 to DL3 induced by plasmid and RL1 to RL3 induced by v2_DEN2) with heterozygous/biallelic/homozygous C-to-T conversions at *OsACC-T33* target identified by Sanger sequencing. The four C bases shown in red at the wild-type (WT) sequence is situated in the deamination window. The T bases marked in red at the six mutant sequences are the results of C-to-T conversion by A3A-PBE. (c) Sanger sequencing chromatograms of six representative rice mutants as in (b).


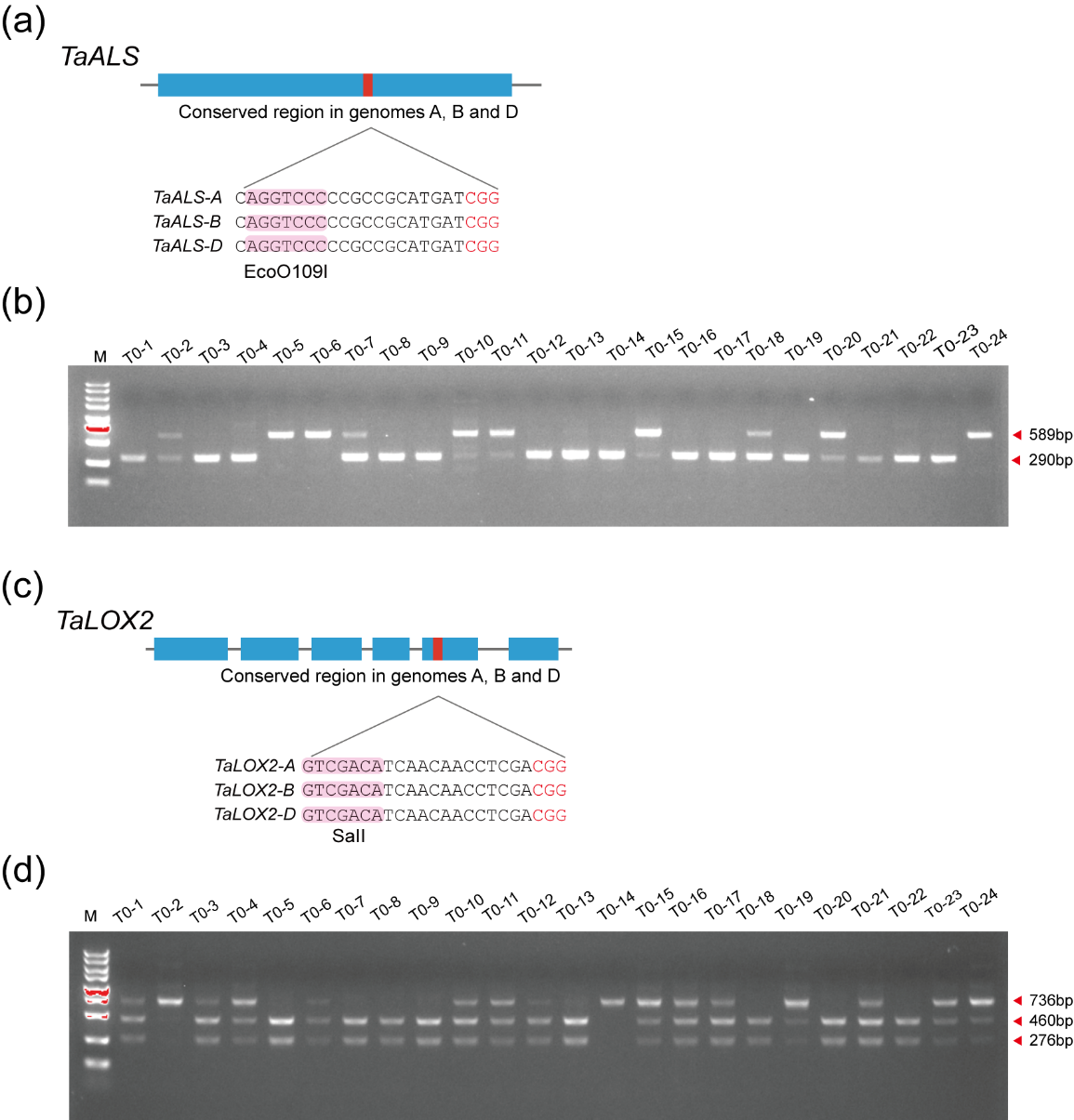


**Figure S5** The outcome of PCR-RE assays for representative wheat mutants. (a) Sequence of the sgRNA targeting a conserved region of the CDS of *TaALS* homoeologous. The PAM sequence is highlighted in red and the EcoO109I restriction site is highlighted in light pink. (b) PCR-RE analyses of 24 representative wheat T0 lines. Lanes T0-1 to T0-24 show PCR fragments amplified from independent wheat plants that digested with EcoO109I. The bands marked by 589bp indicate positive base editing. (c) Sequence of the sgRNA targeting a conserved region of exon 5 of *TaLOX2*. The PAM sequence is highlighted in red and the Sall restriction site is highlighted in light pink. (d) PCR-RE analyses of 24 representative wheat T0 lines. Lanes T0-1 to T0-24 show PCR fragments amplified from independent wheat plants that digested with Sall. The bands marked by 736bp indicate positive base editing.


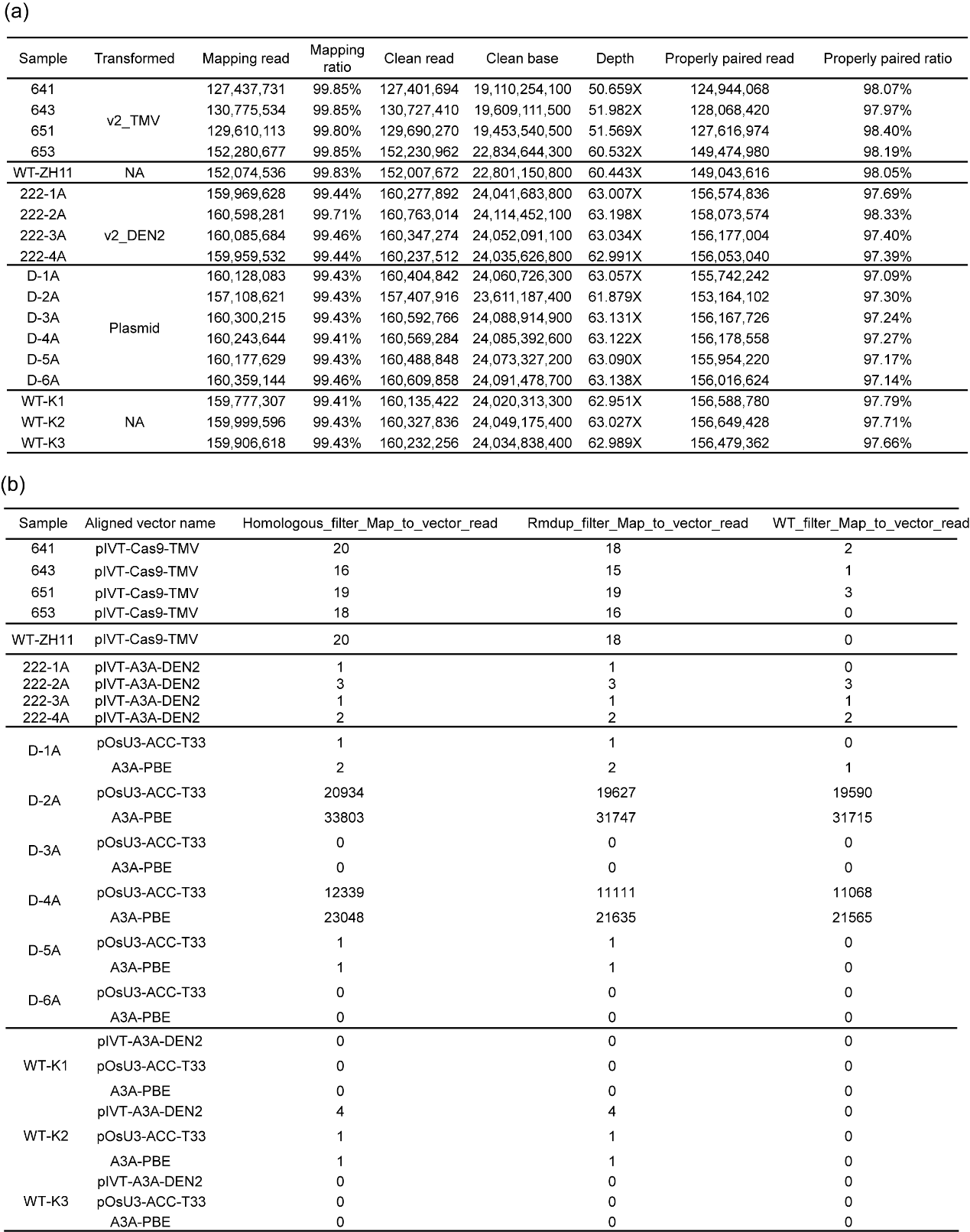


**Figure S6** The description of the whole genome sequencing data **(a)** and the analysis of exogenous DNA fragment insertion in mutants obtained via plasmid- or mRNA-based delivery system **(b)**. NA, not available. The variety of 641, 643, 651 and 653 is ZH11. WT-ZH11 represent the wild type control for rice variety ZH11. The variety of 222-1A, 222-2A, 222-3A, 222-4A, D-1A, D-2A D-3A, D-4A, D-5A and D-6A is Kitaake. WT-K1, WT-K2 and WT-K3 represent the wild type control for rice variety Kitaake. NA, not available.

**Supplementary tables**

**Table S1.** Sequences of 5’UTRs used in this study.

| Name | Sequence | Annotation and accession number |
| --- | --- | --- |
| OsRbsC | ACCACAGCCAGAGCCCGGGTCGAGATGCCACCACGGCCACAATCCACGAGCCCGGCGCGACACCACCGCGCGCGCGTGAGCCAGCCACAAACGCCCGCGGATAGGCGCGCGCACGCCGGCCAATCCTACCACATCCCCGGCCTCCGCGGCTCGCGAGCGCCGCTGCCATCCGATCCGCTGAGTTTTGGCTATTTATACGTACCGCGGGAGCCTGTGTGCAGAGCAGTGCATCTCAAGAAGTACTCGAGCAAAGAAGGAGAGAGCTTGGTGAGCTGCAGAG | *Oryza sativa* small subunit of ribulose-1,5-bisphosphate carboxylase,  AB067656.1 |
| CYBA | GGGCGCGCCTAGCAGTGTCCCAGCCGGGTTCGTGTCGCC | *Homo sapiens* cytochrome b-245 alpha chain, NM_000101.4 |
| OsActin | CCCTCCCTCCGCTTCCAAAGAAACGCCCCCCATCGCCACTATATACATACCCCCCCcTCTCCTCCCATCCCCCcAACCCTACCACCACCACCACCACCACCTCCACCTCCTCCCCCCTCGCTGCCGGACGACGAGCTCCTCCCCCCTCCCCCTCCGCCGCCGCCGCGCCGGTAGAAG | *Oryza sativa* actin gene, EU155408.1 |
| ZmCDK | CGCCGAACGGCAGTGCTGCAAAAGTCCGGACAAAGCTCGCGCCGCCCACCGCACCCGATCCGCCTCGCCCAGGCGTGCTGCCGTCCGCTCCCTTCTCCCGCGCCGTCGCCTCCGCCTACGCCCGGGCCGACCCGCTGGACCCAGCGTGCGCCGCTCCACTCGGACGCGCCGCCGCCCTCTCGGGTTGAGGCGGAGGTGCTTGGGGACCCGCCCCC | *Zea mays* CDC2+/CDC28-related protein kinase, EU967414.1 |
| OsGAPDH | GGAACCTTCTGGAAACCCACGCCCTCTCTATCCCCTCTCCCAATCCAGCGACGTGGCCGCATCTCCCACCGTCCACTTCCCCCCTCCCGATTCAAACCAACGGCCCCGATCCCATCCTTGAGCCGCGGGCACAGAGCCCTGGATCCCCCGGCGGAGCCAGTTCTATATAAAGGCGAGGCCGCCCCAGCTGGCACCACCCATTCCTCGCGTCGCATCGCACTTGTAGCTCTCGACCCCCGCATCTCATCCCTCCTCTCGCTTAGTTCAGATCGAAATCGCAA | *Oryza sativa* glyceraldehyde-3-phosphate dehydrogenase 2, NM_001402381.1 |
| OsUbi1 | TCGCATCAGCTCCACCCCCGAAAAATTTCTCCCCAATCTCGCGAGGCTCTCGTCGTCGAATCGAATCCTCTCGCGTCCTCAAG | *Oryza sativa* polyubiquitin2, AF184280.1 |
| OsUbi3.3 | CCTCCGTCAATCGAACCCCAAAATCGCAGAGAAAAAAAAATCTCCCCTCGAAGCGAAGCGTCGAATCGCCTTCTCAAG | *Oryza sativa* polyubiquitin3 transcript 3, AF184279.1 |
| OsUbi3.2 | ATGCGATTTTCTGATCCTCTCCGTTCCTCGCGTTTGATTTGATTTCCCGGCCTGTTCGTGATTGTGAGATGTTGTG | *Oryza sativa* polyubiquitin3 transcript 2, AF184279.1 |
| OsEF1A | CAAAGATCGGACGGCCAGGAAGGAACCCTAGCACCCGTGGCTGCCTATATAAGAGGCAATCCTCTCGCCCTAACCCCCTCCTCTCTCCCATCTCATCCCAGCCGCCGCCGCCGCAGCCTCCTCCTCTCGCCATCTCCCGTCCTCTCCTTCAAGTCAGCTACCTGCTTAATCAACC | *Oryza sativa* elongation factor 1-alpha, XM_015774251.3 |
| HBB | ACATTTGCTTCTGACACAACTGTGTTCACTAGCAACCTCAAACAGACACC | *Homo sapiens* hemoglobin subunit beta, LC507571.1 |
| CMV1 | GTTTATTTACAAGAGCGTACGGTTCAATCCCTGCCTCCCCTGTAAAACTACCCTTTGAAAACCTCTCTTTCTTAATCTTTTCTTTGTAATTCC | Cucumber mosaic virus RNA 1, NC_002034.1 |
| CMV2 | GTTTATTTACAAGAGCGTACGGTTCAACCCCTGCCTCCCCTGTAAAACTCCCTAGACTTAAATCTTTTCTTTCTAGTATCTTTTC | Cucumber mosaic virus RNA 2, NC_002035.1 |
| OsEIF4A.1 | GGAACGGGAAGGGGGAGACGAGACGAGGAGAAGAGAAGAGAGAAGAGGTCGAGCCGCGCTCCGCCTCCGTCGGGTCCCCTTTATAACCTCTCTCTCTCTTTCTCTCTCTCTCCTCCCAAACCCTCAATCGTCGCATCGCCTCCTCCTCCTCTCGCCGCTCGCAGCGAAAAAGCCAGCGCCTCCCAGCGCCCACGCGATCCCGACGAATCGAATCGAGTCCGAGACGCCGATCTCGGATTCGAGACGGTTGATTTCAACCAACACAAGCCCTCCTCCGGATCGATCCGCTCCTCTTCAGCC | *Oryza sativa* eukaryotic initiation factor 4A-3-like, NM_001406445.1 |
| OsEIF4A.2 | GTCGGGTCCCCTTTATAACCTCTCTCTCTCTTTCTCTCTCTCTCCTCCCAAACCCTCAATCGTCGCATCGCCTCCTCCTCCTCTCGCCGCTCGCAGCGAAAAAGCCAGCGCCTCCCAGCGCCCACGCGATCCCGACGAATCGAATCGAGTCCGAGACGCCGATCTCGGATTCGAGACGGTTGATTTCAACCAACACAAGCCCTCCTCCGGATCGATCCGCTCCTCTTCAGCC | *Oryza sativa* eukaryotic initiation factor 4A-3-like,variant 1, NM_001406445.1 |
| OsRPS27A | TCTGATTTCGCCTAAACCCTAGCCGTCCACACCCCACCACCACCGCCGTCGCCTCCACCCCGCCGCCGCGGGAGAAACCACAGGTAAGGGCAAGCGCGCCGCCGCTGCGGCCGCCGGCGAAG | *Oryza sativa* ubiquitin-40S ribosomal protein S27a-1-like, NM_001409022. |
| OsE2 | CTCCTCATAACTTCTTCCCCACGGGCCAACGGCTGCGCGTCTTCCCGTTCGTCCTCCTGTCCGGTCCGAGTCCCTCTGACGACGCGACTTTCCCCACGCCGGGATCGAGCTCCGGGGAGGGGAGCTGCGAGCTGAAACACTTTGTCATATCAGTCAACAGTAGAGAGGAGACGAG | *Oryza sativa* ubiquitin-conjugating enzyme, XM_052288300.1 |
| OsRPL24 | TTCCCTACTCCCACCCCCCGCCGCCGCCGCCACCACCGCAGCTGCGCCGCTTCAAGGCAGGACATTGTGAAAGTTTCCCAAGTCTGAAAAG | *Oryza sativa* large ribosomal subunit protein eL24, XM_015765228.3 |
| OsRPL11 | TAGGGTTTCCGCCGCCGAAGCCACCACACCTAAACCCTTCTTCCCTCTCCTCCTCCGCCGCCGCCGCCCTCCCCTCTCGCC | *Oryza sativa* large subunit ribosomal protein L11, NM_001420260.1 |
| OsTubulin | CTCCCCGGCCTCTCCACCCACCCCTCGATCTCTCGCTCGCTCGCCGCCGATCGGATCGCGTGGTTGGATCATCACAACTCTGCAAAG | *Oryza sativa* beta-tubulin, NM_001406352.1 |
| HBA2 | GGGACTCTTCTGGTCCCCACAGACTCAGAGAGAACCCACC | *Homo sapiens* hemoglobin subunit alpha 2, MK600513.1 |
| TMV | GTATTTTTACAACAATTACCAACAACAACAAACAACAAACAACATTACAATTACTATTTACAATTACA | Tobacco mosaic virus |
| PVX | ACACCACCAACACAACCAAACCCACCACGCCCAATTGTTACACACCCGCTTGGAAAAGCAAGTCTAACAA | Potato virus X, MT264741.1 |
| AMV | ACCTCGAGTTTTTATTTTTAATTTTCTTTCAAATACTTCCATCCC | alfalfa mosaic virus |
| BMV | GTAAAAGAAAAGGAACAACCCTGTTGTTGTTCGACGCTATACTAAATATATATTATCTTATTAGTGCATTTCTTTTACCGCTTCACAG | Barley stripe mosaic virus RNA 2 |
| synj | ACACGCTGGAATTCTAGTATACTAAACC | Synthetic 5’UTR from Kanoria and Burma, 2012 |
| DEN2 | AGTTGTTAGTCTACGTGGACCGACAAAGACAGATTCTTTGAGGGAGCTAAGCTCAACGTAGTTCTAACAGTTTTTTAATTAGAGAGCAGATCTCTG | Dengue virus type 2 |

**Table S2.** Description of sgRNA sites and sequences.

| Target site | Target sequence | Oligo-F | Oligo-R |
| --- | --- | --- | --- |
| OsAAT-T1 | CAAGGATCCCAGCCCCGTGA**AGG** | GGCGCAAGGATCCCAGCCCCGTGA | AAACTCACGGGGCTGGGATCCTTG |
| OsALS-T5 | GCGCCCCCACTTGGGATCAT**AGG** | GGCGGCGCCCCCACTTGGGATCAT | AAACATGATCCCAAGTGGGGGCGC |
| OsACC-T33 | GACCAGCCTATTATTCTTAC**AGG** | GGCGGTAAGAATAATAGGCTGGTC | AAACGTAAGAATAATAGGCTGGTC |
| OsCDC48-T3 | TAGCACCCATGACAATGACA**TGG** | GGCGTAGCACCCATGACAATGACA | AAACTGTCATTGTCATGGGTGCTA |
| OsDEP1-T2 | AGCTCAAGGAGGAAATTCAT**GGG** | GGCGAGCTCAAGGAGGAAATTCAT | AAACATGAATTTCCTCCTTGAGCT |
| OsDEP1-T3 | AGCACATGAGAGAACAATAT**TGG** | GGCGAGCACATGAGAGAACAATAT | AAACCCAATATTGTTCTCTCATGTGC |
| OsEVEN | CACACACACACTAGTACCTC**TGG** | GGCGCACACACACACTAGTACCTC | AAACGAGGTACTAGTGTGTGTGTG |
| OsNRT1.1B-T2 | GGCCATGGCGCCCGCGGCGG**CGG** | GGCGGGCCATGGCGCCCGCGGCGG | AAACCCGCCGCGGGCGCCATGGCC |
| OsNRT1.1B-T1 | ACTAGATATCTAAACCATTA**AGG** | GGCGACTAGATATCTAAACCATTA | AAACTAATGGTTTAGATATCTAGT |
| OsOD | ACACACACACTAGTACCTCT**GGG** | GGCGACACACACACTAGTACCTCT | AAACAGAGGTACTAGTGTGTGTGT |
| OsPDS | GTTGGTCTTTGCTCCTGCAG**AGG** | GGCGGTTGGTCTTTGCTCCTGCAG | AAACCTGCAGGAGCAAAGACCAAC |
| OsWxb | TCCTCGGTACGACCAGTACA**AGG** | GGCGTCCTCGGTACGACCAGTACA | AAACTGTACTGGTCGTACCGAGGA |
| TaGW2 | CACAAGAAAATCCACCAGGA**TGG** | CTTGCACAAGAAAATCCACCAGGA | AAACTCCTGGTGGATTTTCTTGTG |
| TaALS | CAGGTCCCCCGCCGCATGAT**CGG** | CTTGCAGGTCCCCCGCCGCATGAT | AAACATCATGCGGCGGGGGACCTG |
| TaDEP1 | ACGAGCTACATTTACTTGAA**GGG** | CTTGACGAGCTACATTTACTTGAA | AAACTTCAAGTAAATGTAGCTCGT |
| TaLOX2-T4 | GTGCCGCGCGACGAGCTCTT**CGG** | CTTGGTGCCGCGCGACGAGCTCTT | AAACAAGAGCTCGTCGCGCGGCAC |
| TaLOX2-T5 | GTCGACATCAACAACCTCGA**CGG** | CTTGGTCGACATCAACAACCTCGA | AAACTCGAGGTTGTTGATGTCGAC |

The PAMs in each target sequence are shown in bold.

**Table S3.** PCR primers used in this study.

| Primer names | Primer sequences (5’-3’) | applications |
| --- | --- | --- |
| OsAAT-F | AGGTTAAGTACGCTGGTGCGC | 1 st round PCR for deep sequencing |
| OsAAT-R | GACGATTCAAAGCAAGAATGGTGCC |  |
| OsACC-F | ACGTTCAGAGCTGGATCATTTGGCC |  |
| OsACC-R | AACGGTGTTGTTACTGGAAGTGGTCC |  |
| OsALS-F | ATAGGGCGCATACATACTTG |  |
| OsALS-R | TGATATCTTGTGATGCATATGCCT |  |
| OsCDC48-F | TCTTTCTGATTAATGGCCCGGAG |  |
| OsCDC48-R | GGATGCATTGGAGAGCAGCC |  |
| OsCDC48PE-T1T2T3-F | TATGCTTGCGTGCTAACGGAG |  |
| OsCDC48PE-T1T2T3-R | GTGTCTCGCCGACCAAATGAAG |  |
| OsDEP1-F | AGGTCAGTCCCTGCTTTGGTACCCG |  |
| OsDEP1-R | ACTGCCATTTTGGAGGATCTAAACAGGGCC |  |
| OsEVEN-F | GGGAGATGAGAGAGCTTGTGCC |  |
| OsEVEN-R | GAGTAGTGTAGTACTGAAGAAGCACAGC |  |
| OsGAPDH-F | GTGTGGTGTGCTCACGATTG |  |
| OsGAPDH-R | CTTGTGGCACATCTCTGCCTAC |  |
| OsNRT1.1B-T1-F | TGCTATGTTACGAGATGGAGCG |  |
| OsNRT1.1B-T1-R | TCATCTTCACTTAGCAACTCCTACTC |  |
| OsNRT1.1B-T2-F | TCTTCGTGGGCCTCTCCCTCTC |  |
| OsNRT1.1B-T2-R | ATACGTGCGATGAGAATACACCCAA |  |
| OsWxb-F | ATGAACGTCGTGTTCGTCGG |  |
| OsWxb-R | TTCTCCAGGAATGACGGATGGTCG |  |
| OsOD-F | GGGAGATGAGAGAGCTTGTGCC |  |
| OsOD-R | GAGTAGTGTAGTACTGAAGAAGCACAGC |  |
| OsPDS-F | AGTGCATGTGTTACAGAAGGAATCC |  |
| OsPDS-R | GCTAGATAGAACCCTTCAATCGGTG |  |
| TaGW2-F | AGAATTGTTGAGATGCAATTTATCTTCTAAACTG |  |
| TaGW2-R | TCCTTCCTCTCTTACCACTTCCC |  |
| TaALS-F | GCGCCGACATCCTCGTCGAG |  |
| TaALS-R | CCAACCAGACGCAGGACCTGCTCAA |  |
| TaDEP1-F | GAGGTCCTACATAATATTTACAGGACCAATTCTCTGG |  |
| TaDEP1-R | GGTACGACTTACAGACTAATCCATACATATGGC |  |
| TaLOX2-F | CGTCTACCGCTACGACGTCTACAACG |  |
| TaLOX2-R | GGTCGCCGTACTTGCTCGGATCAAGT |  |
| TaLOX2-T5-F | ACTCCGTCTACCGACCATTGAGC |  |
| TaLOX2-T5-R | TAGACCATGGAGGACATGGGCATGG |  |
| OsDEP1T2-V0-F1 | GTAGAGGCATAGTCTATTTTACTTCAACAGGTAG | 2nd PCR for deep sequencing in **Fig.2** |
| OsDEP1T2-V0-R1 | GTCCGCGTAGTACATACTCTTTAATGCATCC |  |
| OsDEP1T2-V0-F2 | GTTTCGGCATAGTCTATTTTACTTCAACAGGTAG |  |
| OsDEP1T2-V0-R2 | CGTACGGTAGTACATACTCTTTAATGCATCC |  |
| OsDEP1T2-V0-F3 | GAGTGGGCATAGTCTATTTTACTTCAACAGGTAG |  |
| OsDEP1T2-V0-R3 | GGTAGCGTAGTACATACTCTTTAATGCATCC |  |
| OsDEP1T2-V1-F1 | ACTGATCATAGTCTATTTTACTTCAACAGGTAG |  |
| OsDEP1T2-V1-R1 | ATGAGCGTAGTACATACTCTTTAATGCATCC |  |
| OsDEP1T2-V1-F2 | ATTCCTCATAGTCTATTTTACTTCAACAGGTAG |  |
| OsDEP1T2-V1-R2 | CAAAAGGTAGTACATACTCTTTAATGCATCC |  |
| OsDEP1T2-V1-F3 | CACGATCATAGTCTATTTTACTTCAACAGGTAG |  |
| OsDEP1T2-V1-R3 | CACTCAGTAGTACATACTCTTTAATGCATCC |  |
| OsWxb-V0-F1 | ATTCCTTGTTCTTGATCATCGCATTGG |  |
| OsWxb-V0-R1 | CAAAAGGTAACAAATCATCTAAAGCTAATCG |  |
| OsWxb-V0-F2 | GTTTCGTGTTCTTGATCATCGCATTGG |  |
| OsWxb-V0-R2 | CGTACGGTAACAAATCATCTAAAGCTAATCG |  |
| OsWxb-V0-F3 | GAGTGGTGTTCTTGATCATCGCATTGG |  |
| OsWxb-V0-R3 | GGTAGCGTAACAAATCATCTAAAGCTAATCG |  |
| OsWxb-V1-F1 | GTAGAGTGTTCTTGATCATCGCATTGG |  |
| OsWxb-V1-R1 | GTCCGCGTAACAAATCATCTAAAGCTAATCG |  |
| OsWxb-V1-F2 | ACTGATTGTTCTTGATCATCGCATTGG |  |
| OsWxb-V1-R2 | ATGAGCGTAACAAATCATCTAAAGCTAATCG |  |
| OsWxb-V1-F3 | CACGATTGTTCTTGATCATCGCATTGG |  |
| OsWxb-V1-R3 | CACTCAGTAACAAATCATCTAAAGCTAATCG |  |
| OsALST5-V0-F1 | CGATGTCCGCCATCAAGAAGATGC |  |
| OsALST5-V0-R1 | TGACCATTCAGGTCAAACATAGGCCG |  |
| OsALST5-V0-F2 | ACAGTGCCGCCATCAAGAAGATGC |  |
| OsALST5-V0-R2 | GCCAATTTCAGGTCAAACATAGGCCG |  |
| OsALST5-V0-F3 | CAGATCCCGCCATCAAGAAGATGC |  |
| OsALST5-V0-R3 | CTTGTATTCAGGTCAAACATAGGCCG |  |
| OsALST5-V1-F1 | ATTCCTCCGCCATCAAGAAGATGC |  |
| OsALST5-V1-R1 | CAAAAGTTCAGGTCAAACATAGGCCG |  |
| OsALST5-V1-F2 | CAGGCGCCGCCATCAAGAAGATGC |  |
| OsALST5-V1-R2 | CATGGCTTCAGGTCAAACATAGGCCG |  |
| OsALST5-V1-F3 | CATTTTCCGCCATCAAGAAGATGC |  |
| OsALST5-V1-R3 | CCAACATTCAGGTCAAACATAGGCCG |  |
| OsALST5-DNA-F1 | GCGCTACCGCCATCAAGAAGATGC |  |
| OsCDC48T3-V0-F1 | ACAGTGAAGTTGAGAGGCGCATCG |  |
| OsCDC48T3-V0-R1 | GCCAATCATCAGGAACACCAATGTCAATC |  |
| OsCDC48T3-V0-F2 | CAGATCAAGTTGAGAGGCGCATCG |  |
| OsCDC48T3-V0-R2 | CTTGTACATCAGGAACACCAATGTCAATC |  |
| OsCDC48T3-V0-F3 | ATCACGAAGTTGAGAGGCGCATCG |  |
| OsCDC48T3-V0-R3 | TTAGGCCATCAGGAACACCAATGTCAATC |  |
| OsCDC48T3-V1-F1 | ACTTGAAAGTTGAGAGGCGCATCG |  |
| OsCDC48T3-V1-R1 | GATCAGCATCAGGAACACCAATGTCAATC |  |
| OsCDC48T3-V1-F2 | TAGCTTAAGTTGAGAGGCGCATCG |  |
| OsCDC48T3-V1-R2 | GGCTACCATCAGGAACACCAATGTCAATC |  |
| OsCDC48T3-V1-F3 | GAGTGGAAGTTGAGAGGCGCATCG |  |
| OsCDC48T3-V1-R3 | GGTAGCCATCAGGAACACCAATGTCAATC |  |
| GL-Cas9-F1 | CAGGCGCATAGTCTATTTTACTTCAACAGGTAG |  |
| GL-Cas9-R1 | CATGGCGTAGTACATACTCTTTAATGCATCC |  |
| GL-Cas9-F2 | CATTTTCATAGTCTATTTTACTTCAACAGGTAG |  |
| GL-Cas9-R2 | CCAACAGTAGTACATACTCTTTAATGCATCC |  |
| GL-Cas9-F3 | CGGAATGCATAGTCTATTTTACTTCAACAGGTAG |  |
| GL-Cas9-R3 | CTAGCTGTAGTACATACTCTTTAATGCATCC |  |
| HB-Cas9-F1 | ACTGATCATAGTCTATTTTACTTCAACAGGTAG |  |
| HB-Cas9-R1 | ATGAGCGTAGTACATACTCTTTAATGCATCC |  |
| HB-Cas9-F2 | ATTCCTCATAGTCTATTTTACTTCAACAGGTAG |  |
| HB-Cas9-R2 | CAAAAGGTAGTACATACTCTTTAATGCATCC |  |
| HB-Cas9-F3 | CACGATCATAGTCTATTTTACTTCAACAGGTAG |  |
| HB-Cas9-R3 | CACTCAGTAGTACATACTCTTTAATGCATCC |  |
| DEN-Cas9-F1 | GTAGAGGCATAGTCTATTTTACTTCAACAGGTAG |  |
| DEN-Cas9-R1 | GTCCGCGTAGTACATACTCTTTAATGCATCC |  |
| DEN-Cas9-F2 | GTGAAAGCATAGTCTATTTTACTTCAACAGGTAG |  |
| DEN-Cas9-R2 | GTGGCCGTAGTACATACTCTTTAATGCATCC |  |
| DEN-Cas9-F3 | GAGTGGGCATAGTCTATTTTACTTCAACAGGTAG |  |
| DEN-Cas9-R3 | GGTAGCGTAGTACATACTCTTTAATGCATCC |  |
| EF1a-Cas9-F1 | ACTTGAGCATAGTCTATTTTACTTCAACAGGTAG |  |
| EF1a-Cas9-R1 | GATCAGGTAGTACATACTCTTTAATGCATCC |  |
| EF1a-Cas9-F2 | AGTCAAGCATAGTCTATTTTACTTCAACAGGTAG |  |
| EF1a-Cas9-R2 | AGTTCCGTAGTACATACTCTTTAATGCATCC |  |
| EF1a-Cas9-F3 | ATGTCAGCATAGTCTATTTTACTTCAACAGGTAG |  |
| EF1a-Cas9-R3 | CCGTCCGTAGTACATACTCTTTAATGCATCC |  |
| TMV1-Cas9-F1 | CGATGTGCATAGTCTATTTTACTTCAACAGGTAG |  |
| TMV1-Cas9-R1 | TGACCAGTAGTACATACTCTTTAATGCATCC |  |
| TMV1-Cas9-F2 | ACAGTGGCATAGTCTATTTTACTTCAACAGGTAG |  |
| TMV1-Cas9-R2 | GCCAATGTAGTACATACTCTTTAATGCATCC |  |
| TMV1-Cas9-F3 | ATCACGGCATAGTCTATTTTACTTCAACAGGTAG |  |
| TMV1-Cas9-R3 | TTAGGCGTAGTACATACTCTTTAATGCATCC |  |
| SEF-Cas9-F1 | CGATGTGCATAGTCTATTTTACTTCAACAGGTAG |  |
| SEF-Cas9-R1 | TGACCAGTAGTACATACTCTTTAATGCATCC |  |
| SEF-Cas9-F2 | ACAGTGGCATAGTCTATTTTACTTCAACAGGTAG |  |
| SEF-Cas9-R2 | GCCAATGTAGTACATACTCTTTAATGCATCC |  |
| SEF-Cas9-F3 | CAGATCGCATAGTCTATTTTACTTCAACAGGTAG |  |
| SEF-Cas9-R3 | CTTGTAGTAGTACATACTCTTTAATGCATCC |  |
| LEF-Cas9-F1 | GTAGAGGCATAGTCTATTTTACTTCAACAGGTAG |  |
| LEF-Cas9-R1 | GTCCGCGTAGTACATACTCTTTAATGCATCC |  |
| LEF-Cas9-F2 | GTTTCGGCATAGTCTATTTTACTTCAACAGGTAG |  |
| LEF-Cas9-R2 | CGTACGGTAGTACATACTCTTTAATGCATCC |  |
| LEF-Cas9-F3 | GAGTGGGCATAGTCTATTTTACTTCAACAGGTAG |  |
| LEF-Cas9-R3 | GGTAGCGTAGTACATACTCTTTAATGCATCC |  |
| RPS-Cas9-F1 | GCGCTAGCATAGTCTATTTTACTTCAACAGGTAG |  |
| RPS-Cas9-R1 | TAATCGGTAGTACATACTCTTTAATGCATCC |  |
| RPS-Cas9-F2 | TACAGCGCATAGTCTATTTTACTTCAACAGGTAG |  |
| RPS-Cas9-R2 | TATAATGTAGTACATACTCTTTAATGCATCC |  |
| RPS-Cas9-F3 | TCGAAGGCATAGTCTATTTTACTTCAACAGGTAG |  |
| RPS-Cas9-R3 | TCGGCAGTAGTACATACTCTTTAATGCATCC |  |
| ACTIN-Cas9-F1 | CATTTTCATAGTCTATTTTACTTCAACAGGTAG |  |
| ACTIN-Cas9-R1 | CCAACAGTAGTACATACTCTTTAATGCATCC |  |
| ACTIN-Cas9-F2 | CGGAATGCATAGTCTATTTTACTTCAACAGGTAG |  |
| ACTIN-Cas9-R2 | CTAGCTGTAGTACATACTCTTTAATGCATCC |  |
| ACTIN-Cas9-F3 | CTATACGCATAGTCTATTTTACTTCAACAGGTAG |  |
| ACTIN-Cas9-R3 | CTCAGAGTAGTACATACTCTTTAATGCATCC |  |
| E2-Cas9-F1 | ACTGATCATAGTCTATTTTACTTCAACAGGTAG |  |
| E2-Cas9-R1 | ATGAGCGTAGTACATACTCTTTAATGCATCC |  |
| E2-Cas9-F2 | ATTCCTCATAGTCTATTTTACTTCAACAGGTAG |  |
| E2-Cas9-R2 | CAAAAGGTAGTACATACTCTTTAATGCATCC |  |
| E2-Cas9-F3 | CAACTACATAGTCTATTTTACTTCAACAGGTAG |  |
| E2-Cas9-R3 | CACCGGGTAGTACATACTCTTTAATGCATCC |  |
| TMV2-Cas9-F1 | ACTTGAGCATAGTCTATTTTACTTCAACAGGTAG |  |
| TMV2-Cas9-R1 | GATCAGGTAGTACATACTCTTTAATGCATCC |  |
| TMV2-Cas9-F2 | TAGCTTGCATAGTCTATTTTACTTCAACAGGTAG |  |
| TMV2-Cas9-R2 | GGCTACGTAGTACATACTCTTTAATGCATCC |  |
| TMV2-Cas9-F3 | ATGTCAGCATAGTCTATTTTACTTCAACAGGTAG |  |
| TMV2-Cas9-R3 | CCGTCCGTAGTACATACTCTTTAATGCATCC |  |
| AMV-Cas9-F1 | CGATGTGCATAGTCTATTTTACTTCAACAGGTAG |  |
| AMV-Cas9-R1 | TGACCAGTAGTACATACTCTTTAATGCATCC |  |
| AMV-Cas9-F2 | CAGATCGCATAGTCTATTTTACTTCAACAGGTAG |  |
| AMV-Cas9-R2 | CTTGTAGTAGTACATACTCTTTAATGCATCC |  |
| AMV-Cas9-F3 | ATCACGGCATAGTCTATTTTACTTCAACAGGTAG |  |
| AMV-Cas9-R3 | TTAGGCGTAGTACATACTCTTTAATGCATCC |  |
| BMV-Cas9-F1 | TAGCTTGCATAGTCTATTTTACTTCAACAGGTAG |  |
| BMV-Cas9-R1 | GGCTACGTAGTACATACTCTTTAATGCATCC |  |
| BMV-Cas9-F2 | AGTCAAGCATAGTCTATTTTACTTCAACAGGTAG |  |
| BMV-Cas9-R2 | AGTTCCGTAGTACATACTCTTTAATGCATCC |  |
| BMV-Cas9-F3 | ATGTCAGCATAGTCTATTTTACTTCAACAGGTAG |  |
| BMV-Cas9-R3 | CCGTCCGTAGTACATACTCTTTAATGCATCC |  |
| PVX-Cas9-F1 | TACAGCGCATAGTCTATTTTACTTCAACAGGTAG |  |
| PVX-Cas9-R1 | TATAATGTAGTACATACTCTTTAATGCATCC |  |
| PVX-Cas9-F2 | TCATTCGCATAGTCTATTTTACTTCAACAGGTAG |  |
| PVX-Cas9-R2 | TCCCGAGTAGTACATACTCTTTAATGCATCC |  |
| PVX-Cas9-F3 | TCGAAGGCATAGTCTATTTTACTTCAACAGGTAG |  |
| PVX-Cas9-R3 | TCGGCAGTAGTACATACTCTTTAATGCATCC |  |
| TMV3-Cas9-F1 | CAGGCGCATAGTCTATTTTACTTCAACAGGTAG |  |
| TMV3-Cas9-R1 | CATGGCGTAGTACATACTCTTTAATGCATCC |  |
| TMV3-Cas9-F2 | CATTTTCATAGTCTATTTTACTTCAACAGGTAG |  |
| TMV3-Cas9-R2 | CCAACAGTAGTACATACTCTTTAATGCATCC |  |
| TMV3-Cas9-F3 | CTATACGCATAGTCTATTTTACTTCAACAGGTAG |  |
| TMV3-Cas9-R3 | CTCAGAGTAGTACATACTCTTTAATGCATCC |  |
| U1-Cas9-F1 | CGATGTGCATAGTCTATTTTACTTCAACAGGTAG |  |
| U1-Cas9-R1 | TGACCAGTAGTACATACTCTTTAATGCATCC |  |
| U1-Cas9-F2 | ACAGTGGCATAGTCTATTTTACTTCAACAGGTAG |  |
| U1-Cas9-R2 | GCCAATGTAGTACATACTCTTTAATGCATCC |  |
| U1-Cas9-F3 | CAGATCGCATAGTCTATTTTACTTCAACAGGTAG |  |
| U1-Cas9-R3 | CTTGTAGTAGTACATACTCTTTAATGCATCC |  |
| U2-Cas9-F1 | ACTTGAGCATAGTCTATTTTACTTCAACAGGTAG |  |
| U2-Cas9-R1 | GATCAGGTAGTACATACTCTTTAATGCATCC |  |
| U2-Cas9-F2 | TAGCTTGCATAGTCTATTTTACTTCAACAGGTAG |  |
| U2-Cas9-R2 | GGCTACGTAGTACATACTCTTTAATGCATCC |  |
| U2-Cas9-F3 | AGTCAAGCATAGTCTATTTTACTTCAACAGGTAG |  |
| U2-Cas9-R3 | AGTTCCGTAGTACATACTCTTTAATGCATCC |  |
| U3-Cas9-F1 | GTAGAGGCATAGTCTATTTTACTTCAACAGGTAG |  |
| U3-Cas9-R1 | GTCCGCGTAGTACATACTCTTTAATGCATCC |  |
| U3-Cas9-F2 | GTGAAAGCATAGTCTATTTTACTTCAACAGGTAG |  |
| U3-Cas9-R2 | GTGGCCGTAGTACATACTCTTTAATGCATCC |  |
| U3-Cas9-F3 | GTTTCGGCATAGTCTATTTTACTTCAACAGGTAG |  |
| U3-Cas9-R3 | CGTACGGTAGTACATACTCTTTAATGCATCC |  |
| RBP-Cas9-F1 | ACTGATCATAGTCTATTTTACTTCAACAGGTAG |  |
| RBP-Cas9-R1 | ATGAGCGTAGTACATACTCTTTAATGCATCC |  |
| RBP-Cas9-F2 | ATTCCTCATAGTCTATTTTACTTCAACAGGTAG |  |
| RBP-Cas9-R2 | CAAAAGGTAGTACATACTCTTTAATGCATCC |  |
| RBP-Cas9-F3 | CAACTACATAGTCTATTTTACTTCAACAGGTAG |  |
| RBP-Cas9-R3 | CACCGGGTAGTACATACTCTTTAATGCATCC |  |
| TUB-Cas9-F1 | CATTTTCATAGTCTATTTTACTTCAACAGGTAG |  |
| TUB-Cas9-R1 | CCAACAGTAGTACATACTCTTTAATGCATCC |  |
| TUB-Cas9-F2 | CGGAATGCATAGTCTATTTTACTTCAACAGGTAG |  |
| TUB-Cas9-R2 | CTAGCTGTAGTACATACTCTTTAATGCATCC |  |
| TUB-Cas9-F3 | CTATACGCATAGTCTATTTTACTTCAACAGGTAG |  |
| TUB-Cas9-R3 | CTCAGAGTAGTACATACTCTTTAATGCATCC |  |
| TMV4-Cas9-F1 | GCGCTAGCATAGTCTATTTTACTTCAACAGGTAG |  |
| TMV4-Cas9-R1 | TAATCGGTAGTACATACTCTTTAATGCATCC |  |
| TMV4-Cas9-F2 | TACAGCGCATAGTCTATTTTACTTCAACAGGTAG |  |
| TMV4-Cas9-R2 | TATAATGTAGTACATACTCTTTAATGCATCC |  |
| TMV4-Cas9-F3 | TCATTCGCATAGTCTATTTTACTTCAACAGGTAG |  |
| TMV4-Cas9-R3 | TCCCGAGTAGTACATACTCTTTAATGCATCC |  |
| AG-Cas9-F1 | ACAGTGGCATAGTCTATTTTACTTCAACAGGTAG |  |
| AG-Cas9-R1 | GCCAATGTAGTACATACTCTTTAATGCATCC |  |
| AG-Cas9-F2 | CAGATCGCATAGTCTATTTTACTTCAACAGGTAG |  |
| AG-Cas9-R2 | CTTGTAGTAGTACATACTCTTTAATGCATCC |  |
| AG-Cas9-F3 | ATCACGGCATAGTCTATTTTACTTCAACAGGTAG |  |
| AG-Cas9-R3 | TTAGGCGTAGTACATACTCTTTAATGCATCC |  |
| CYBA-Cas9-F1 | ACTTGAGCATAGTCTATTTTACTTCAACAGGTAG |  |
| CYBA-Cas9-R1 | GATCAGGTAGTACATACTCTTTAATGCATCC |  |
| CYBA-Cas9-F2 | TAGCTTGCATAGTCTATTTTACTTCAACAGGTAG |  |
| CYBA-Cas9-R2 | GGCTACGTAGTACATACTCTTTAATGCATCC |  |
| CYBA-Cas9-F3 | AGTCAAGCATAGTCTATTTTACTTCAACAGGTAG |  |
| CYBA-Cas9-R3 | AGTTCCGTAGTACATACTCTTTAATGCATCC |  |
| RBSC-Cas9-F1 | GTAGAGGCATAGTCTATTTTACTTCAACAGGTAG |  |
| RBSC-Cas9-R1 | GTCCGCGTAGTACATACTCTTTAATGCATCC |  |
| RBSC-Cas9-F2 | GTGAAAGCATAGTCTATTTTACTTCAACAGGTAG |  |
| RBSC-Cas9-R2 | GTGGCCGTAGTACATACTCTTTAATGCATCC |  |
| RBSC-Cas9-F3 | GTTTCGGCATAGTCTATTTTACTTCAACAGGTAG |  |
| RBSC-Cas9-R3 | CGTACGGTAGTACATACTCTTTAATGCATCC |  |
| RBPL-Cas9-F1 | ACTGATCATAGTCTATTTTACTTCAACAGGTAG |  |
| RBPL-Cas9-R1 | ATGAGCGTAGTACATACTCTTTAATGCATCC |  |
| RBPL-Cas9-F2 | ATTCCTCATAGTCTATTTTACTTCAACAGGTAG |  |
| RBPL-Cas9-R2 | CAAAAGGTAGTACATACTCTTTAATGCATCC |  |
| RBPL-Cas9-F3 | CAACTACATAGTCTATTTTACTTCAACAGGTAG |  |
| RBPL-Cas9-R3 | CACCGGGTAGTACATACTCTTTAATGCATCC |  |
| CDK-Cas9-F1 | CAGGCGCATAGTCTATTTTACTTCAACAGGTAG |  |
| CDK-Cas9-R1 | CATGGCGTAGTACATACTCTTTAATGCATCC |  |
| CDK-Cas9-F2 | CATTTTCATAGTCTATTTTACTTCAACAGGTAG |  |
| CDK-Cas9-R2 | CCAACAGTAGTACATACTCTTTAATGCATCC |  |
| CDK-Cas9-F3 | CGGAATGCATAGTCTATTTTACTTCAACAGGTAG |  |
| CDK-Cas9-R3 | CTAGCTGTAGTACATACTCTTTAATGCATCC |  |
| TMV5-Cas9-F1 | GCGCTAGCATAGTCTATTTTACTTCAACAGGTAG |  |
| TMV5-Cas9-R1 | TAATCGGTAGTACATACTCTTTAATGCATCC |  |
| TMV5-Cas9-F2 | TACAGCGCATAGTCTATTTTACTTCAACAGGTAG |  |
| TMV5-Cas9-R2 | TATAATGTAGTACATACTCTTTAATGCATCC |  |
| TMV5-Cas9-F3 | TCATTCGCATAGTCTATTTTACTTCAACAGGTAG |  |
| TMV5-Cas9-R3 | TCCCGAGTAGTACATACTCTTTAATGCATCC |  |
| CM1-Cas9-F1 | CGATGTGCATAGTCTATTTTACTTCAACAGGTAG |  |
| CM1-Cas9-R1 | TGACCAGTAGTACATACTCTTTAATGCATCC |  |
| CM1-Cas9-F2 | CAGATCGCATAGTCTATTTTACTTCAACAGGTAG |  |
| CM1-Cas9-R2 | CTTGTAGTAGTACATACTCTTTAATGCATCC |  |
| CM1-Cas9-F3 | ATCACGGCATAGTCTATTTTACTTCAACAGGTAG |  |
| CM1-Cas9-R3 | TTAGGCGTAGTACATACTCTTTAATGCATCC |  |
| CM2-Cas9-F1 | ACTTGAGCATAGTCTATTTTACTTCAACAGGTAG |  |
| CM2-Cas9-R1 | GATCAGGTAGTACATACTCTTTAATGCATCC |  |
| CM2-Cas9-F2 | AGTCAAGCATAGTCTATTTTACTTCAACAGGTAG |  |
| CM2-Cas9-R2 | AGTTCCGTAGTACATACTCTTTAATGCATCC |  |
| CM2-Cas9-F3 | ATGTCAGCATAGTCTATTTTACTTCAACAGGTAG |  |
| CM2-Cas9-R3 | CCGTCCGTAGTACATACTCTTTAATGCATCC |  |
| synj-Cas9-F1 | GTAGAGGCATAGTCTATTTTACTTCAACAGGTAG |  |
| synj-Cas9-R1 | GTCCGCGTAGTACATACTCTTTAATGCATCC |  |
| synj-Cas9-F2 | GTTTCGGCATAGTCTATTTTACTTCAACAGGTAG |  |
| synj-Cas9-R2 | CGTACGGTAGTACATACTCTTTAATGCATCC |  |
| synj-Cas9-F3 | GAGTGGGCATAGTCTATTTTACTTCAACAGGTAG |  |
| synj-Cas9-R3 | GGTAGCGTAGTACATACTCTTTAATGCATCC |  |
| TMV6-Cas9-F1 | GCGCTAGCATAGTCTATTTTACTTCAACAGGTAG |  |
| TMV6-Cas9-R1 | TAATCGGTAGTACATACTCTTTAATGCATCC |  |
| TMV6-Cas9-F2 | TCATTCGCATAGTCTATTTTACTTCAACAGGTAG |  |
| TMV6-Cas9-R2 | TCCCGAGTAGTACATACTCTTTAATGCATCC |  |
| TMV6-Cas9-F3 | TCGAAGGCATAGTCTATTTTACTTCAACAGGTAG |  |
| TMV6-Cas9-R3 | TCGGCAGTAGTACATACTCTTTAATGCATCC |  |
| TEV-Cas9-F1 | GTGAAAGCATAGTCTATTTTACTTCAACAGGTAG |  |
| TEV-Cas9-R1 | GTGGCCGTAGTACATACTCTTTAATGCATCC |  |
| TEV-Cas9-F2 | GTTTCGGCATAGTCTATTTTACTTCAACAGGTAG |  |
| TEV-Cas9-R2 | CGTACGGTAGTACATACTCTTTAATGCATCC |  |
| TEV-Cas9-F3 | GAGTGGGCATAGTCTATTTTACTTCAACAGGTAG |  |
| TEV-Cas9-R3 | GGTAGCGTAGTACATACTCTTTAATGCATCC |  |
| TMV7-Cas9-F1 | CGATGTGCATAGTCTATTTTACTTCAACAGGTAG |  |
| TMV7-Cas9-R1 | TGACCAGTAGTACATACTCTTTAATGCATCC |  |
| TMV7-Cas9-F2 | ACAGTGGCATAGTCTATTTTACTTCAACAGGTAG |  |
| TMV7-Cas9-R2 | GCCAATGTAGTACATACTCTTTAATGCATCC |  |
| TMV7-Cas9-F3 | ATCACGGCATAGTCTATTTTACTTCAACAGGTAG |  |
| TMV7-Cas9-R3 | TTAGGCGTAGTACATACTCTTTAATGCATCC |  |
| NAAC-F1 | CGATGTGCATAGTCTATTTTACTTCAACAGGTAG |  |
| NAAC-R1 | TGACCAGTAGTACATACTCTTTAATGCATCC |  |
| NAAC-F2 | ACAGTGGCATAGTCTATTTTACTTCAACAGGTAG |  |
| NAAC-R2 | GCCAATGTAGTACATACTCTTTAATGCATCC |  |
| NAAC-F3 | CAGATCGCATAGTCTATTTTACTTCAACAGGTAG |  |
| NAAC-R3 | CTTGTAGTAGTACATACTCTTTAATGCATCC |  |
| PRO-F1 | ATCACGGCATAGTCTATTTTACTTCAACAGGTAG |  |
| PRO-R1 | TTAGGCGTAGTACATACTCTTTAATGCATCC |  |
| PRO-F2 | ACTTGAGCATAGTCTATTTTACTTCAACAGGTAG |  |
| PRO-R2 | GATCAGGTAGTACATACTCTTTAATGCATCC |  |
| PRO-F3 | TAGCTTGCATAGTCTATTTTACTTCAACAGGTAG |  |
| PRO-R3 | GGCTACGTAGTACATACTCTTTAATGCATCC |  |
| TRANS2020-F1 | AGTCAAGCATAGTCTATTTTACTTCAACAGGTAG |  |
| TRANS2020-R1 | AGTTCCGTAGTACATACTCTTTAATGCATCC |  |
| TRANS2020-F2 | ATGTCAGCATAGTCTATTTTACTTCAACAGGTAG |  |
| TRANS2020-R2 | CCGTCCGTAGTACATACTCTTTAATGCATCC |  |
| TRANS2020-F3 | GTAGAGGCATAGTCTATTTTACTTCAACAGGTAG |  |
| TRANS2020-R3 | GTCCGCGTAGTACATACTCTTTAATGCATCC |  |
| TRANSRNA-F1 | GTGAAAGCATAGTCTATTTTACTTCAACAGGTAG |  |
| TRANSRNA-R1 | GTGGCCGTAGTACATACTCTTTAATGCATCC |  |
| TRANSRNA-F2 | GTTTCGGCATAGTCTATTTTACTTCAACAGGTAG |  |
| TRANSRNA-R2 | CGTACGGTAGTACATACTCTTTAATGCATCC |  |
| TRANSRNA-F3 | GAGTGGGCATAGTCTATTTTACTTCAACAGGTAG |  |
| TRANSRNA-R3 | GGTAGCGTAGTACATACTCTTTAATGCATCC |  |
| LIPO-F1 | ACTGATCATAGTCTATTTTACTTCAACAGGTAG |  |
| LIPO-R1 | ATGAGCGTAGTACATACTCTTTAATGCATCC |  |
| LIPO-F2 | ATTCCTCATAGTCTATTTTACTTCAACAGGTAG |  |
| LIPO-R2 | CAAAAGGTAGTACATACTCTTTAATGCATCC |  |
| LIPO-F3 | CAACTACATAGTCTATTTTACTTCAACAGGTAG |  |
| LIPO-R3 | CACCGGGTAGTACATACTCTTTAATGCATCC |  |
| JET-F1 | CACGATCATAGTCTATTTTACTTCAACAGGTAG |  |
| JET-R1 | CACTCAGTAGTACATACTCTTTAATGCATCC |  |
| JET-F2 | CAGGCGCATAGTCTATTTTACTTCAACAGGTAG |  |
| JET-R2 | CATGGCGTAGTACATACTCTTTAATGCATCC |  |
| JET-F3 | CATTTTCATAGTCTATTTTACTTCAACAGGTAG |  |
| JET-R3 | CCAACAGTAGTACATACTCTTTAATGCATCC |  |
| PRO-0-F1 | CGATGTGCATAGTCTATTTTACTTCAACAGGTAG |  |
| PRO-0-R1 | TGACCAGTAGTACATACTCTTTAATGCATCC |  |
| PRO-0-F2 | CAGATCGCATAGTCTATTTTACTTCAACAGGTAG |  |
| PRO-0-R2 | CTTGTAGTAGTACATACTCTTTAATGCATCC |  |
| PRO-0-F3 | ATCACGGCATAGTCTATTTTACTTCAACAGGTAG |  |
| PRO-0-R3 | TTAGGCGTAGTACATACTCTTTAATGCATCC |  |
| PRO-0.5-F1 | ACTTGAGCATAGTCTATTTTACTTCAACAGGTAG |  |
| PRO-0.5-R1 | GATCAGGTAGTACATACTCTTTAATGCATCC |  |
| PRO-0.5-F2 | AGTCAAGCATAGTCTATTTTACTTCAACAGGTAG |  |
| PRO-0.5-R2 | AGTTCCGTAGTACATACTCTTTAATGCATCC |  |
| PRO-0.5-F3 | ATGTCAGCATAGTCTATTTTACTTCAACAGGTAG |  |
| PRO-0.5-R3 | CCGTCCGTAGTACATACTCTTTAATGCATCC |  |
| PRO-1-F1 | GTAGAGGCATAGTCTATTTTACTTCAACAGGTAG |  |
| PRO-1-R1 | GTCCGCGTAGTACATACTCTTTAATGCATCC |  |
| PRO-1-F2 | GTGAAAGCATAGTCTATTTTACTTCAACAGGTAG |  |
| PRO-1-R2 | GTGGCCGTAGTACATACTCTTTAATGCATCC |  |
| PRO-1-F3 | GAGTGGGCATAGTCTATTTTACTTCAACAGGTAG |  |
| PRO-1-R3 | GGTAGCGTAGTACATACTCTTTAATGCATCC |  |
| PRO-1.5-F1 | ACTGATCATAGTCTATTTTACTTCAACAGGTAG |  |
| PRO-1.5-R1 | ATGAGCGTAGTACATACTCTTTAATGCATCC |  |
| PRO-1.5-F2 | ATTCCTCATAGTCTATTTTACTTCAACAGGTAG |  |
| PRO-1.5-R2 | CAAAAGGTAGTACATACTCTTTAATGCATCC |  |
| PRO-1.5-F3 | CAACTACATAGTCTATTTTACTTCAACAGGTAG |  |
| PRO-1.5-R3 | CACCGGGTAGTACATACTCTTTAATGCATCC |  |
| PRO-2.5-F1 | CAGGCGCATAGTCTATTTTACTTCAACAGGTAG |  |
| PRO-2.5-R1 | CATGGCGTAGTACATACTCTTTAATGCATCC |  |
| PRO-2.5-F2 | CATTTTCATAGTCTATTTTACTTCAACAGGTAG |  |
| PRO-2.5-R2 | CCAACAGTAGTACATACTCTTTAATGCATCC |  |
| PRO-2.5-F3 | CGGAATGCATAGTCTATTTTACTTCAACAGGTAG |  |
| PRO-2.5-R3 | CTAGCTGTAGTACATACTCTTTAATGCATCC |  |
| PRO-3.5-F1 | TACAGCGCATAGTCTATTTTACTTCAACAGGTAG |  |
| PRO-3.5-R1 | TATAATGTAGTACATACTCTTTAATGCATCC |  |
| PRO-3.5-F2 | TCATTCGCATAGTCTATTTTACTTCAACAGGTAG |  |
| PRO-3.5-R2 | TCCCGAGTAGTACATACTCTTTAATGCATCC |  |
| PRO-3.5-F3 | TCGAAGGCATAGTCTATTTTACTTCAACAGGTAG |  |
| PRO-3.5-R3 | TCGGCAGTAGTACATACTCTTTAATGCATCC |  |
| LOX2PRO-0-F1 | TAGCTTACTTGGTCTGACGATCCGCATG |  |
| LOX2PRO-0-R1 | GGCTACAGGTGCGGGTGGCTGGTATG |  |
| LOX2PRO-0-F2 | AGTCAAACTTGGTCTGACGATCCGCATG |  |
| LOX2PRO-0-R2 | AGTTCCAGGTGCGGGTGGCTGGTATG |  |
| LOX2PRO-0-F3 | ATGTCAACTTGGTCTGACGATCCGCATG |  |
| LOX2PRO-0-R3 | CCGTCCAGGTGCGGGTGGCTGGTATG |  |
| LOX2PRO-1-F1 | GTAGAGACTTGGTCTGACGATCCGCATG |  |
| LOX2PRO-1-R1 | GTCCGCAGGTGCGGGTGGCTGGTATG |  |
| LOX2PRO-1-F2 | GTTTCGACTTGGTCTGACGATCCGCATG |  |
| LOX2PRO-1-R2 | CGTACGAGGTGCGGGTGGCTGGTATG |  |
| LOX2PRO-1-F3 | GAGTGGACTTGGTCTGACGATCCGCATG |  |
| LOX2PRO-1-R3 | GGTAGCAGGTGCGGGTGGCTGGTATG |  |
| LOX2PRO-1.5-F1 | ATTCCTACTTGGTCTGACGATCCGCATG |  |
| LOX2PRO-1.5-R1 | CAAAAGAGGTGCGGGTGGCTGGTATG |  |
| LOX2PRO-1.5-F2 | CAACTAACTTGGTCTGACGATCCGCATG |  |
| LOX2PRO-1.5-R2 | CACCGGAGGTGCGGGTGGCTGGTATG |  |
| LOX2PRO-1.5-F3 | CACGATACTTGGTCTGACGATCCGCATG |  |
| LOX2PRO-1.5-R3 | CACTCAAGGTGCGGGTGGCTGGTATG |  |
| LOX2PRO-2.5-F1 | CAGGCGACTTGGTCTGACGATCCGCATG |  |
| LOX2PRO-2.5-R1 | CATGGCAGGTGCGGGTGGCTGGTATG |  |
| LOX2PRO-2.5-F2 | CATTTTACTTGGTCTGACGATCCGCATG |  |
| LOX2PRO-2.5-R2 | CCAACAAGGTGCGGGTGGCTGGTATG |  |
| LOX2PRO-2.5-F3 | CTATACACTTGGTCTGACGATCCGCATG |  |
| LOX2PRO-2.5-R3 | CTCAGAAGGTGCGGGTGGCTGGTATG |  |
| LOX2PRO-3.5-F1 | GCGCTAACTTGGTCTGACGATCCGCATG |  |
| LOX2PRO-3.5-R1 | TAATCGAGGTGCGGGTGGCTGGTATG |  |
| LOX2PRO-3.5-F2 | TCATTCACTTGGTCTGACGATCCGCATG |  |
| LOX2PRO-3.5-R2 | TCCCGAAGGTGCGGGTGGCTGGTATG |  |
| LOX2PRO-3.5-F3 | TCGAAGACTTGGTCTGACGATCCGCATG |  |
| LOX2PRO-3.5-R3 | TCGGCAAGGTGCGGGTGGCTGGTATG |  |
| MgPRO-0-F1 | ATCACGGATACTGTACGTCGACGAGG |  |
| MgPRO-0-R1 | AAGTACTGACGGTACCGACCTGTGCG |  |
| MgPRO-0-F2 | CGATGTGATACTGTACGTCGACGAGG |  |
| MgPRO-0-R2 | TGCCATTGACGGTACCGACCTGTGCG |  |
| MgPRO-0-F3 | TGACCAGATACTGTACGTCGACGAGG |  |
| MgPRO-0-R3 | AGATAGTGACGGTACCGACCTGTGCG |  |
| MgPRO-1-F1 | TAGCTTGATACTGTACGTCGACGAGG |  |
| MgPRO-1-R1 | GCAGGATGACGGTACCGACCTGTGCG |  |
| MgPRO-1-F2 | GGCTACGATACTGTACGTCGACGAGG |  |
| MgPRO-1-R2 | GCCGCGTGACGGTACCGACCTGTGCG |  |
| MgPRO-1-F3 | CTTGTAGATACTGTACGTCGACGAGG |  |
| MgPRO-1-R3 | AGCGCATGACGGTACCGACCTGTGCG |  |
| MgPRO-1.5-F1 | AGTTCCGATACTGTACGTCGACGAGG |  |
| MgPRO-1.5-R1 | ATCGTCTGACGGTACCGACCTGTGCG |  |
| MgPRO-1.5-F2 | ATGTCAGATACTGTACGTCGACGAGG |  |
| MgPRO-1.5-R2 | CCGCAATGACGGTACCGACCTGTGCG |  |
| MgPRO-1.5-F3 | CCGTCCGATACTGTACGTCGACGAGG |  |
| MgPRO-1.5-R3 | AACTTGTGACGGTACCGACCTGTGCG |  |
| MgPRO-2.5-F1 | GTAGAGGATACTGTACGTCGACGAGG |  |
| MgPRO-2.5-R1 | AAGGTATGACGGTACCGACCTGTGCG |  |
| MgPRO-2.5-F2 | GTCCGCGATACTGTACGTCGACGAGG |  |
| MgPRO-2.5-R2 | GCGCTCTGACGGTACCGACCTGTGCG |  |
| MgPRO-2.5-F3 | GTGGCCGATACTGTACGTCGACGAGG |  |
| MgPRO-2.5-R3 | ACGATTTGACGGTACCGACCTGTGCG |  |
| MgPRO-3.5-F1 | GTTTCGGATACTGTACGTCGACGAGG |  |
| MgPRO-3.5-R1 | AGACTCTGACGGTACCGACCTGTGCG |  |
| MgPRO-3.5-F2 | CGTACGGATACTGTACGTCGACGAGG |  |
| MgPRO-3.5-R2 | TGGTCTTGACGGTACCGACCTGTGCG |  |
| MgPRO-3.5-F3 | GAGTGGGATACTGTACGTCGACGAGG |  |
| MgPRO-3.5-R3 | GGAATTTGACGGTACCGACCTGTGCG |  |
| ABE-EVENTMV-F1 | CGATGTCCTTGCTTTCATTCTTCAGTGC | 2nd PCR for deep sequencing in **Fig.3** |
| ABE-EVENTMV-R1 | TGACCAGCAGACCAAGATCCCAAGAAC |  |
| ABE-EVENTMV-F2 | ACAGTGCCTTGCTTTCATTCTTCAGTGC |  |
| ABE-EVENTMV-R2 | GCCAATGCAGACCAAGATCCCAAGAAC |  |
| ABE-EVENTMV-F3 | ATCACGCCTTGCTTTCATTCTTCAGTGC |  |
| ABE-EVENTMV-R3 | TTAGGCGCAGACCAAGATCCCAAGAAC |  |
| ABE-EVENDEN-F1 | ACTTGACCTTGCTTTCATTCTTCAGTGC |  |
| ABE-EVENDEN-R1 | GATCAGGCAGACCAAGATCCCAAGAAC |  |
| ABE-EVENDEN-F2 | TAGCTTCCTTGCTTTCATTCTTCAGTGC |  |
| ABE-EVENDEN-R2 | GGCTACGCAGACCAAGATCCCAAGAAC |  |
| ABE-EVENDEN-F3 | AGTCAACCTTGCTTTCATTCTTCAGTGC |  |
| ABE-EVENDEN-R3 | AGTTCCGCAGACCAAGATCCCAAGAAC |  |
| ABE-EVENDNA-F1 | ATTCCTCCTTGCTTTCATTCTTCAGTGC |  |
| ABE-EVENDNA-R1 | CAAAAGGCAGACCAAGATCCCAAGAAC |  |
| ABE-EVENDNA-F2 | CAACTACCTTGCTTTCATTCTTCAGTGC |  |
| ABE-EVENDNA-R2 | CACCGGGCAGACCAAGATCCCAAGAAC |  |
| ABE-EVENDNA-F3 | CACGATCCTTGCTTTCATTCTTCAGTGC |  |
| ABE-EVENDNA-R3 | CACTCAGCAGACCAAGATCCCAAGAAC |  |
| ABE-ODTMV-F1 | ACTTGACCTTGCTTTCATTCTTCAGTGC |  |
| ABE-ODTMV-R1 | GATCAGGCAGACCAAGATCCCAAGAAC |  |
| ABE-ODTMV-F2 | TAGCTTCCTTGCTTTCATTCTTCAGTGC |  |
| ABE-ODTMV-R2 | GGCTACGCAGACCAAGATCCCAAGAAC |  |
| ABE-ODTMV-F3 | AGTCAACCTTGCTTTCATTCTTCAGTGC |  |
| ABE-ODTMV-R3 | AGTTCCGCAGACCAAGATCCCAAGAAC |  |
| ABE-ODDEN-F1 | ACAGTGCCTTGCTTTCATTCTTCAGTGC |  |
| ABE-ODDEN-R1 | GCCAATGCAGACCAAGATCCCAAGAAC |  |
| ABE-ODDEN-F2 | CAGATCCCTTGCTTTCATTCTTCAGTGC |  |
| ABE-ODDEN-R2 | CTTGTAGCAGACCAAGATCCCAAGAAC |  |
| ABE-ODDEN-F3 | ATCACGCCTTGCTTTCATTCTTCAGTGC |  |
| ABE-ODDEN-R3 | TTAGGCGCAGACCAAGATCCCAAGAAC |  |
| ABE-ODDNA-F1 | GTAGAGCCTTGCTTTCATTCTTCAGTGC |  |
| ABE-ODDNA-R1 | GTCCGCGCAGACCAAGATCCCAAGAAC |  |
| ABE-ODDNA-F2 | GTGAAACCTTGCTTTCATTCTTCAGTGC |  |
| ABE-ODDNA-R2 | GTGGCCGCAGACCAAGATCCCAAGAAC |  |
| ABE-ODDNA-F3 | GAGTGGCCTTGCTTTCATTCTTCAGTGC |  |
| ABE-ODDNA-R3 | GGTAGCGCAGACCAAGATCCCAAGAAC |  |
| ABE-NRTTMV-F1 | CGATGTCACTCCAGCCACTCACTGTC |  |
| ABE-NRTTMV-R1 | TGACCACGACTCTAGCTATATACCTGGCAG |  |
| ABE-NRTTMV-F2 | ACAGTGCACTCCAGCCACTCACTGTC |  |
| ABE-NRTTMV-R2 | GCCAATCGACTCTAGCTATATACCTGGCAG |  |
| ABE-NRTTMV-F3 | CAGATCCACTCCAGCCACTCACTGTC |  |
| ABE-NRTTMV-R3 | CTTGTACGACTCTAGCTATATACCTGGCAG |  |
| ABE-NRTDEN-F1 | ACTTGACACTCCAGCCACTCACTGTC |  |
| ABE-NRTDEN-R1 | GATCAGCGACTCTAGCTATATACCTGGCAG |  |
| ABE-NRTDEN-F2 | TAGCTTCACTCCAGCCACTCACTGTC |  |
| ABE-NRTDEN-R2 | GGCTACCGACTCTAGCTATATACCTGGCAG |  |
| ABE-NRTDEN-F3 | AGTCAACACTCCAGCCACTCACTGTC |  |
| ABE-NRTDEN-R3 | AGTTCCCGACTCTAGCTATATACCTGGCAG |  |
| ABE-NRTDNA-F1 | GTAGAGCACTCCAGCCACTCACTGTC |  |
| ABE-NRTDNA-R1 | GTCCGCCGACTCTAGCTATATACCTGGCAG |  |
| ABE-NRTDNA-F2 | GTGAAACACTCCAGCCACTCACTGTC |  |
| ABE-NRTDNA-R2 | GTGGCCCGACTCTAGCTATATACCTGGCAG |  |
| ABE-NRTDNA-F3 | GAGTGGCACTCCAGCCACTCACTGTC |  |
| ABE-NRTDNA-R3 | GGTAGCCGACTCTAGCTATATACCTGGCAG |  |
| ABE-DEP1TMV-F1 | ACTTGAACCTACAGTGGGTGACTTTTGACAT |  |
| ABE-DEP1TMV-R1 | GATCAGGTACGACTTACAGACTAATCCATACATATGGC |  |
| ABE-DEP1TMV-F2 | TAGCTTACCTACAGTGGGTGACTTTTGACAT |  |
| ABE-DEP1TMV-R2 | GGCTACGTACGACTTACAGACTAATCCATACATATGGC |  |
| ABE-DEP1TMV-F3 | ATGTCAACCTACAGTGGGTGACTTTTGACAT |  |
| ABE-DEP1TMV-R3 | CCGTCCGTACGACTTACAGACTAATCCATACATATGGC |  |
| ABE-DEP1DEN-F1 | CGATGTACCTACAGTGGGTGACTTTTGACAT |  |
| ABE-DEP1DEN-R1 | TGACCAGTACGACTTACAGACTAATCCATACATATGGC |  |
| ABE-DEP1DEN-F2 | ACAGTGACCTACAGTGGGTGACTTTTGACAT |  |
| ABE-DEP1DEN-R2 | GCCAATGTACGACTTACAGACTAATCCATACATATGGC |  |
| ABE-DEP1DEN-F3 | CAGATCACCTACAGTGGGTGACTTTTGACAT |  |
| ABE-DEP1DEN-R3 | CTTGTAGTACGACTTACAGACTAATCCATACATATGGC |  |
| ABE-DEP1DNA-F1 | ACTGATACCTACAGTGGGTGACTTTTGACAT |  |
| ABE-DEP1DNA-R1 | ATGAGCGTACGACTTACAGACTAATCCATACATATGGC |  |
| ABE-DEP1DNA-F2 | CAACTAACCTACAGTGGGTGACTTTTGACAT |  |
| ABE-DEP1DNA-R2 | CACCGGGTACGACTTACAGACTAATCCATACATATGGC |  |
| ABE-DEP1DNA-F3 | CACGATACCTACAGTGGGTGACTTTTGACAT |  |
| ABE-DEP1DNA-R3 | CACTCAGTACGACTTACAGACTAATCCATACATATGGC |  |
| ABE-GW2TMV-F1 | GTAGAGGCAGGAGCAAGGAAGTATAGGAAATC |  |
| ABE-GW2TMV-R1 | GTCCGCGCGGAACATGTCAAAGACTGGG |  |
| ABE-GW2TMV-F2 | GTGAAAGCAGGAGCAAGGAAGTATAGGAAATC |  |
| ABE-GW2TMV-R2 | GTGGCCGCGGAACATGTCAAAGACTGGG |  |
| ABE-GW2TMV-F3 | GAGTGGGCAGGAGCAAGGAAGTATAGGAAATC |  |
| ABE-GW2TMV-R3 | GGTAGCGCGGAACATGTCAAAGACTGGG |  |
| ABE-GW2DEN-F1 | CGATGTGCAGGAGCAAGGAAGTATAGGAAATC |  |
| ABE-GW2DEN-R1 | TGACCAGCGGAACATGTCAAAGACTGGG |  |
| ABE-GW2DEN-F2 | ACAGTGGCAGGAGCAAGGAAGTATAGGAAATC |  |
| ABE-GW2DEN-R2 | GCCAATGCGGAACATGTCAAAGACTGGG |  |
| ABE-GW2DEN-F3 | ATCACGGCAGGAGCAAGGAAGTATAGGAAATC |  |
| ABE-GW2DEN-R3 | TTAGGCGCGGAACATGTCAAAGACTGGG |  |
| ABE-GW2DNA-F1 | ACTTGAGCAGGAGCAAGGAAGTATAGGAAATC |  |
| ABE-GW2DNA-R1 | GATCAGGCGGAACATGTCAAAGACTGGG |  |
| ABE-GW2DNA-F2 | TAGCTTGCAGGAGCAAGGAAGTATAGGAAATC |  |
| ABE-GW2DNA-R2 | GGCTACGCGGAACATGTCAAAGACTGGG |  |
| ABE-GW2DNA-F3 | AGTCAAGCAGGAGCAAGGAAGTATAGGAAATC |  |
| ABE-GW2DNA-R3 | AGTTCCGCGGAACATGTCAAAGACTGGG |  |
| ABE-LOX2T5TMV-F1 | ACTTGAAGGGCCTCACCGTGGAGCAG |  |
| ABE-LOX2T5TMV-R1 | GATCAGTCCCCTCGCAGGAAGAGCAGG |  |
| ABE-LOX2T5TMV-F2 | AGTCAAAGGGCCTCACCGTGGAGCAG |  |
| ABE-LOX2T5TMV-R2 | AGTTCCTCCCCTCGCAGGAAGAGCAGG |  |
| ABE-LOX2T5TMV-F3 | ATGTCAAGGGCCTCACCGTGGAGCAG |  |
| ABE-LOX2T5TMV-R3 | CCGTCCTCCCCTCGCAGGAAGAGCAGG |  |
| ABE-LOX2T5DEN-F1 | CTATACAGGGCCTCACCGTGGAGCAG |  |
| ABE-LOX2T5DEN-R1 | CTCAGATCCCCTCGCAGGAAGAGCAGG |  |
| ABE-LOX2T5DEN-F2 | CAGATCAGGGCCTCACCGTGGAGCAG |  |
| ABE-LOX2T5DEN-R2 | CTTGTATCCCCTCGCAGGAAGAGCAGG |  |
| ABE-LOX2T5DEN-F3 | ATCACGAGGGCCTCACCGTGGAGCAG |  |
| ABE-LOX2T5DEN-R3 | TTAGGCTCCCCTCGCAGGAAGAGCAGG |  |
| ABE-LOX2T5DNA-F1 | GTGAAAAGGGCCTCACCGTGGAGCAG |  |
| ABE-LOX2T5DNA-R1 | GTGGCCTCCCCTCGCAGGAAGAGCAGG |  |
| ABE-LOX2T5DNA-F2 | GTTTCGAGGGCCTCACCGTGGAGCAG |  |
| ABE-LOX2T5DNA-R2 | CGTACGTCCCCTCGCAGGAAGAGCAGG |  |
| ABE-LOX2T5DNA-F3 | GAGTGGAGGGCCTCACCGTGGAGCAG |  |
| ABE-LOX2T5DNA-R3 | GGTAGCTCCCCTCGCAGGAAGAGCAGG |  |
| CBE-CDC48TMV-F1 | ATTCCTAAGTTGAGAGGCGCATCG |  |
| CBE-CDC48TMV-R1 | CAAAAGCATCAGGAACACCAATGTCAATC |  |
| CBE-CDC48TMV-F2 | CAACTAAAGTTGAGAGGCGCATCG |  |
| CBE-CDC48TMV-R2 | CACCGGCATCAGGAACACCAATGTCAATC |  |
| CBE-CDC48TMV-F3 | CACGATAAGTTGAGAGGCGCATCG |  |
| CBE-CDC48TMV-R3 | CACTCACATCAGGAACACCAATGTCAATC |  |
| CBE-CDC48DEN-F1 | CATTTTAAGTTGAGAGGCGCATCG |  |
| CBE-CDC48DEN-R1 | CCAACACATCAGGAACACCAATGTCAATC |  |
| CBE-CDC48DEN-F2 | CGGAATAAGTTGAGAGGCGCATCG |  |
| CBE-CDC48DEN-R2 | CTAGCTCATCAGGAACACCAATGTCAATC |  |
| CBE-CDC48DEN-F3 | CTATACAAGTTGAGAGGCGCATCG |  |
| CBE-CDC48DEN-R3 | CTCAGACATCAGGAACACCAATGTCAATC |  |
| CBE-CDC48DNA-F1 | TGTACGAAGTTGAGAGGCGCATCG |  |
| CBE-CDC48DNA-R1 | ACCAGTCATCAGGAACACCAATGTCAATC |  |
| CBE-CDC48DNA-F2 | GTGACAAAGTTGAGAGGCGCATCG |  |
| CBE-CDC48DNA-R2 | TAACCGCATCAGGAACACCAATGTCAATC |  |
| CBE-CDC48DNA-F3 | GCACTAAAGTTGAGAGGCGCATCG |  |
| CBE-CDC48DNA-R3 | CGCTATCATCAGGAACACCAATGTCAATC |  |
| CBE-WxbTMV-F1 | ACTGATTGTTCTTGATCATCGCATTGG |  |
| CBE-WxbTMV-R1 | ATGAGCGTAACAAATCATCTAAAGCTAATCG |  |
| CBE-WxbTMV-F2 | ATTCCTTGTTCTTGATCATCGCATTGG |  |
| CBE-WxbTMV-R2 | CAAAAGGTAACAAATCATCTAAAGCTAATCG |  |
| CBE-WxbTMV-F3 | CAACTATGTTCTTGATCATCGCATTGG |  |
| CBE-WxbTMV-R3 | CACCGGGTAACAAATCATCTAAAGCTAATCG |  |
| CBE-WxbDEN-F1 | CAGGCGTGTTCTTGATCATCGCATTGG |  |
| CBE-WxbDEN-R1 | CATGGCGTAACAAATCATCTAAAGCTAATCG |  |
| CBE-WxbDEN-F2 | CGGAATTGTTCTTGATCATCGCATTGG |  |
| CBE-WxbDEN-R2 | CTAGCTGTAACAAATCATCTAAAGCTAATCG |  |
| CBE-WxbDEN-F3 | CTATACTGTTCTTGATCATCGCATTGG |  |
| CBE-WxbDEN-R3 | CTCAGAGTAACAAATCATCTAAAGCTAATCG |  |
| CBE-WxbDNA-F1 | TGTACGTGTTCTTGATCATCGCATTGG |  |
| CBE-WxbDNA-R1 | ACCAGTGTAACAAATCATCTAAAGCTAATCG |  |
| CBE-WxbDNA-F2 | GTGACATGTTCTTGATCATCGCATTGG |  |
| CBE-WxbDNA-R2 | TAACCGGTAACAAATCATCTAAAGCTAATCG |  |
| CBE-WxbDNA-F3 | CTAGACTGTTCTTGATCATCGCATTGG |  |
| CBE-WxbDNA-R3 | ATGTTCGTAACAAATCATCTAAAGCTAATCG |  |
| CBE-LOXT5TMV-F1 | ACAGTGAGGGCCTCACCGTGGAGCAG |  |
| CBE-LOXT5TMV-R1 | GCCAATTCCCCTCGCAGGAAGAGCAGG |  |
| CBE-LOXT5TMV-F2 | CAGATCAGGGCCTCACCGTGGAGCAG |  |
| CBE-LOXT5TMV-R2 | CTTGTATCCCCTCGCAGGAAGAGCAGG |  |
| CBE-LOXT5TMV-F3 | ATCACGAGGGCCTCACCGTGGAGCAG |  |
| CBE-LOXT5TMV-R3 | TTAGGCTCCCCTCGCAGGAAGAGCAGG |  |
| CBE-LOXT5DEN-F1 | ACTTGAAGGGCCTCACCGTGGAGCAG |  |
| CBE-LOXT5DEN-R1 | GATCAGTCCCCTCGCAGGAAGAGCAGG |  |
| CBE-LOXT5DEN-F2 | AGTCAAAGGGCCTCACCGTGGAGCAG |  |
| CBE-LOXT5DEN-R2 | AGTTCCTCCCCTCGCAGGAAGAGCAGG |  |
| CBE-LOXT5DEN-F3 | ATGTCAAGGGCCTCACCGTGGAGCAG |  |
| CBE-LOXT5DEN-R3 | CCGTCCTCCCCTCGCAGGAAGAGCAGG |  |
| CBE-LOXT5DNA-F1 | GTGAAAAGGGCCTCACCGTGGAGCAG |  |
| CBE-LOXT5DNA-R1 | GTGGCCTCCCCTCGCAGGAAGAGCAGG |  |
| CBE-LOXT5DNA-F2 | GTTTCGAGGGCCTCACCGTGGAGCAG |  |
| CBE-LOXT5DNA-R2 | CGTACGTCCCCTCGCAGGAAGAGCAGG |  |
| CBE-LOXT5DNA-F3 | GAGTGGAGGGCCTCACCGTGGAGCAG |  |
| CBE-LOXT5DNA-R3 | GGTAGCTCCCCTCGCAGGAAGAGCAGG |  |
| CBE-LOX2TMV-F1 | CGATGTACTTGGTCTGACGATCCGCATG |  |
| CBE-LOX2TMV-R1 | TGACCAAGGTGCGGGTGGCTGGTATG |  |
| CBE-LOX2TMV-F2 | ACAGTGACTTGGTCTGACGATCCGCATG |  |
| CBE-LOX2TMV-R2 | GCCAATAGGTGCGGGTGGCTGGTATG |  |
| CBE-LOX2TMV-F3 | ATCACGACTTGGTCTGACGATCCGCATG |  |
| CBE-LOX2TMV-R3 | TTAGGCAGGTGCGGGTGGCTGGTATG |  |
| CBE-LOX2DEN-F1 | ACTTGAACTTGGTCTGACGATCCGCATG |  |
| CBE-LOX2DEN-R1 | GATCAGAGGTGCGGGTGGCTGGTATGZHEG |  |
| CBE-LOX2DEN-F2 | TAGCTTACTTGGTCTGACGATCCGCATG |  |
| CBE-LOX2DEN-R2 | GGCTACAGGTGCGGGTGGCTGGTATG |  |
| CBE-LOX2DEN-F3 | ATGTCAACTTGGTCTGACGATCCGCATG |  |
| CBE-LOX2DEN-R3 | CCGTCCAGGTGCGGGTGGCTGGTATG |  |
| CBE-LOX2DNA-F1 | GTAGAGACTTGGTCTGACGATCCGCATG |  |
| CBE-LOX2DNA-R1 | GTCCGCAGGTGCGGGTGGCTGGTATG |  |
| CBE-LOX2DNA-F2 | GTGAAAACTTGGTCTGACGATCCGCATG |  |
| CBE-LOX2DNA-R2 | GTGGCCAGGTGCGGGTGGCTGGTATG |  |
| CBE-LOX2DNA-F3 | GAGTGGACTTGGTCTGACGATCCGCATG |  |
| CBE-LOX2DNA-R3 | GGTAGCAGGTGCGGGTGGCTGGTATG |  |
| Cas9-PDSTMV-F1 | CGATGTGAACTCTTTACAGGAATACTATGATCC |  |
| Cas9-PDSTMV-R1 | TGACCACCTCACCTTGGTGTCTTCAC |  |
| Cas9-PDSTMV-F2 | ACAGTGGAACTCTTTACAGGAATACTATGATCC |  |
| Cas9-PDSTMV-R2 | GCCAATCCTCACCTTGGTGTCTTCAC |  |
| Cas9-PDSTMV-F3 | CAGATCGAACTCTTTACAGGAATACTATGATCC |  |
| Cas9-PDSTMV-R3 | CTTGTACCTCACCTTGGTGTCTTCAC |  |
| Cas9-PDSDEN-F1 | ACTTGAGAACTCTTTACAGGAATACTATGATCC |  |
| Cas9-PDSDEN-R1 | GATCAGCCTCACCTTGGTGTCTTCAC |  |
| Cas9-PDSDEN-F2 | TAGCTTGAACTCTTTACAGGAATACTATGATCC |  |
| Cas9-PDSDEN-R2 | GGCTACCCTCACCTTGGTGTCTTCAC |  |
| Cas9-PDSDEN-F3 | ATGTCAGAACTCTTTACAGGAATACTATGATCC |  |
| Cas9-PDSDEN-R3 | CCGTCCCCTCACCTTGGTGTCTTCAC |  |
| Cas9-PDSDNA-F1 | GTAGAGGAACTCTTTACAGGAATACTATGATCC |  |
| Cas9-PDSDNA-R1 | GTCCGCCCTCACCTTGGTGTCTTCAC |  |
| Cas9-PDSDNA-F2 | GTGAAAGAACTCTTTACAGGAATACTATGATCC |  |
| Cas9-PDSDNA-R2 | GTGGCCCCTCACCTTGGTGTCTTCAC |  |
| Cas9-PDSDNA-F3 | GTTTCGGAACTCTTTACAGGAATACTATGATCC |  |
| Cas9-PDSDNA-R3 | CGTACGCCTCACCTTGGTGTCTTCAC |  |
| Cas9-NRTTMV-F1 | CGATGTCACTCCAGCCACTCACTGTC |  |
| Cas9-NRTTMV-R1 | TGACCACGACTCTAGCTATATACCTGGCAG |  |
| Cas9-NRTTMV-F2 | ACAGTGCACTCCAGCCACTCACTGTC |  |
| Cas9-NRTTMV-R2 | GCCAATCGACTCTAGCTATATACCTGGCAG |  |
| Cas9-NRTTMV-F3 | CAGATCCACTCCAGCCACTCACTGTC |  |
| Cas9-NRTTMV-R3 | CTTGTACGACTCTAGCTATATACCTGGCAG |  |
| Cas9-NRTDEN-F1 | ACTTGACACTCCAGCCACTCACTGTC |  |
| Cas9-NRTDEN-R1 | GATCAGCGACTCTAGCTATATACCTGGCAG |  |
| Cas9-NRTDEN-F2 | TAGCTTCACTCCAGCCACTCACTGTC |  |
| Cas9-NRTDEN-R2 | GGCTACCGACTCTAGCTATATACCTGGCAG |  |
| Cas9-NRTDEN-F3 | AGTCAACACTCCAGCCACTCACTGTC |  |
| Cas9-NRTDEN-R3 | AGTTCCCGACTCTAGCTATATACCTGGCAG |  |
| Cas9-NRTDNA-F1 | GTAGAGCACTCCAGCCACTCACTGTC |  |
| Cas9-NRTDNA-R1 | GTCCGCCGACTCTAGCTATATACCTGGCAG |  |
| Cas9-NRTDNA-F2 | GTGAAACACTCCAGCCACTCACTGTC |  |
| Cas9-NRTDNA-R2 | GTGGCCCGACTCTAGCTATATACCTGGCAG |  |
| Cas9-NRTDNA-F3 | GAGTGGCACTCCAGCCACTCACTGTC |  |
| Cas9-NRTDNA-R3 | GGTAGCCGACTCTAGCTATATACCTGGCAG |  |
| Cas9-ACCT33TMV-F1 | ACAGTGAGGAGACATTTACACTTACATTTGTG |  |
| Cas9-ACCT33TMV-R1 | GCCAATTAGTTGCCATGATTTTGGGACC |  |
| Cas9-ACCT33TMV-F2 | CAGATCAGGAGACATTTACACTTACATTTGTG |  |
| Cas9-ACCT33TMV-R2 | CTTGTATAGTTGCCATGATTTTGGGACC |  |
| Cas9-ACCT33TMV-F3 | ATCACGAGGAGACATTTACACTTACATTTGTG |  |
| Cas9-ACCT33TMV-R3 | TTAGGCTAGTTGCCATGATTTTGGGACC |  |
| Cas9-ACCT33DEN-F1 | TAGCTTAGGAGACATTTACACTTACATTTGTG |  |
| Cas9-ACCT33DEN-R1 | GGCTACTAGTTGCCATGATTTTGGGACC |  |
| Cas9-ACCT33DEN-F2 | AGTCAAAGGAGACATTTACACTTACATTTGTG |  |
| Cas9-ACCT33DEN-R2 | AGTTCCTAGTTGCCATGATTTTGGGACC |  |
| Cas9-ACCT33DEN-F3 | ATGTCAAGGAGACATTTACACTTACATTTGTG |  |
| Cas9-ACCT33DEN-R3 | CCGTCCTAGTTGCCATGATTTTGGGACC |  |
| Cas9-ACCT33DNA-F1 | GTAGAGAGGAGACATTTACACTTACATTTGTG |  |
| Cas9-ACCT33DNA-R1 | GTCCGCTAGTTGCCATGATTTTGGGACC |  |
| Cas9-ACCT33DNA-F2 | GTTTCGAGGAGACATTTACACTTACATTTGTG |  |
| Cas9-ACCT33DNA-R2 | CGTACGTAGTTGCCATGATTTTGGGACC |  |
| Cas9-ACCT33DNA-F3 | GAGTGGAGGAGACATTTACACTTACATTTGTG |  |
| Cas9-ACCT33DNA-R3 | GGTAGCTAGTTGCCATGATTTTGGGACC |  |
| Cas9-AAT1TMV-F1 | ACAGTGTCGACCTGATCGGTGCTC |  |
| Cas9-AAT1TMV-R1 | GCCAATATCCACCACCAATCCAATCC |  |
| Cas9-AAT1TMV-F2 | CAGATCTCGACCTGATCGGTGCTC |  |
| Cas9-AAT1TMV-R2 | CTTGTAATCCACCACCAATCCAATCC |  |
| Cas9-AAT1TMV-F3 | ATCACGTCGACCTGATCGGTGCTC |  |
| Cas9-AAT1TMV-R3 | TTAGGCATCCACCACCAATCCAATCC |  |
| Cas9-AAT1DEN-F1 | ACTTGATCGACCTGATCGGTGCTC |  |
| Cas9-AAT1DEN-R1 | GATCAGATCCACCACCAATCCAATCC |  |
| Cas9-AAT1DEN-F2 | TAGCTTTCGACCTGATCGGTGCTC |  |
| Cas9-AAT1DEN-R2 | GGCTACATCCACCACCAATCCAATCC |  |
| Cas9-AAT1DEN-F3 | AGTCAATCGACCTGATCGGTGCTC |  |
| Cas9-AAT1DEN-R3 | AGTTCCATCCACCACCAATCCAATCC |  |
| Cas9-AAT1DNA-F1 | GTAGAGTCGACCTGATCGGTGCTC |  |
| Cas9-AAT1DNA-R1 | GTCCGCATCCACCACCAATCCAATCC |  |
| Cas9-AAT1DNA-F2 | GTGAAATCGACCTGATCGGTGCTC |  |
| Cas9-AAT1DNA-R2 | GTGGCCATCCACCACCAATCCAATCC |  |
| Cas9-AAT1DNA-F3 | GTTTCGTCGACCTGATCGGTGCTC |  |
| Cas9-AAT1DNA-R3 | CGTACGATCCACCACCAATCCAATCC |  |
| Cas9-DEPT3TMV-F1 | ACTTGAGCATAGTCTATTTTACTTCAACAGGTAG |  |
| Cas9-DEPT3TMV-R1 | GATCAGGTAGTACATACTCTTTAATGCATCC |  |
| Cas9-DEPT3TMV-F2 | AGTCAAGCATAGTCTATTTTACTTCAACAGGTAG |  |
| Cas9-DEPT3TMV-R2 | AGTTCCGTAGTACATACTCTTTAATGCATCC |  |
| Cas9-DEPT3TMV-F3 | ATGTCAGCATAGTCTATTTTACTTCAACAGGTAG |  |
| Cas9-DEPT3TMV-R3 | CCGTCCGTAGTACATACTCTTTAATGCATCC |  |
| Cas9-DEPT3DEN-F1 | ACAGTGGCATAGTCTATTTTACTTCAACAGGTAG |  |
| Cas9-DEPT3DEN-R1 | GCCAATGTAGTACATACTCTTTAATGCATCC |  |
| Cas9-DEPT3DEN-F2 | CAGATCGCATAGTCTATTTTACTTCAACAGGTAG |  |
| Cas9-DEPT3DEN-R2 | CTTGTAGTAGTACATACTCTTTAATGCATCC |  |
| Cas9-DEPT3DEN-F3 | ATCACGGCATAGTCTATTTTACTTCAACAGGTAG |  |
| Cas9-DEPT3DEN-R3 | TTAGGCGTAGTACATACTCTTTAATGCATCC |  |
| Cas9-DEPT3DNA-F1 | GTAGAGGCATAGTCTATTTTACTTCAACAGGTAG |  |
| Cas9-DEPT3DNA-R1 | GTCCGCGTAGTACATACTCTTTAATGCATCC |  |
| Cas9-DEPT3DNA-F2 | GTGAAAGCATAGTCTATTTTACTTCAACAGGTAG |  |
| Cas9-DEPT3DNA-R2 | GTGGCCGTAGTACATACTCTTTAATGCATCC |  |
| Cas9-DEPT3DNA-F3 | GAGTGGGCATAGTCTATTTTACTTCAACAGGTAG |  |
| Cas9-DEPT3DNA-R3 | GGTAGCGTAGTACATACTCTTTAATGCATCC |  |
| OsACC-sg33-F | ACGTTCAGAGCTGGATCATTTGGCC | Identification of mutants |
| OsACC-sg33-R | AACGGTGTTGTTACTGGAAGTGGTCC |  |
| OsDEP1T2-F | AGGTCAGTCCCTGCTTTGGTACCCG |  |
| OsDEP1T2-R | ACTGCCATTTTGGAGGATCTAAACAGGGCC |  |
| TaALS-F | GCGCCGACATCCTCGTCGAG |  |
| TaALS-R | CCAACCAGACGCAGGACCTGCTCAA |  |
| TaLOX2T5-F | ACTCCGTCTACCGACCATTGAGC |  |
| TaLOX2T5-R | TAGACCATGGAGGACATGGGCATGG |  |
